# Identification and analysis of long non-coding RNA related miRNA sponge regulatory network in bladder urothelial carcinoma

**DOI:** 10.1186/s12935-019-1052-2

**Published:** 2019-12-03

**Authors:** Jiawu Wang, Chengyao Zhang, Yan Wu, Weiyang He, Xin Gou

**Affiliations:** 1 Department of Urology, The First Affiliated Hospital of Chongqing Medical University, Yuzhong District, Chongqing, China; 2 Department of Head and Neck Cancer Center, Chongqing University Cancer Hospital & Chongqing Cancer Institute & Chongqing Cancer Hospital, Shapingba District, Chongqing, China; 3 Department of General Surgery, University-Town Hospital of Chongqing Medical University, Shapingba District, Chongqing, China

**Keywords:** ceRNA, lncRNA, miRNA, mRNA, Prognosis, Carcinogenesis, BUC

## Abstract

**Background:**

The aim of this study was to investigate the regulatory network of lncRNAs as competing endogenous RNAs (ceRNA) in bladder urothelial carcinoma (BUC) based on gene expression data derived from The Cancer Genome Atlas (TCGA).

**Materials and methods:**

RNA sequence profiles and clinical information from 414 BUC tissues and 19 non-tumor adjacent tissues were downloaded from TCGA. Differentially expressed RNAs derived from BUC and non-tumor adjacent samples were identified using the R package “edgeR”. Kyoto Encyclopedia of Genes and Genomes (KEGG) pathway analysis was performed using the “clusterProfiler” package. Gene ontology and protein–protein interaction (PPI) networks were analyzed for the differentially expressed mRNAs using the “STRING” database. The network for the dysregulated lncRNA associated ceRNAs was then constructed for BUC using miRcode, miRTarBase, miRDB, and TargetScan. Cox regression analysis was performed to identify independent prognostic RNAs associated with BUC overall survival (OS). Survival analysis for the independent prognostic RNAs within the ceRNA network was calculated using Kaplan–Meier curves.

**Results:**

Based on our analysis, a total of 666, 1819 and 157 differentially expressed lncRNAs, mRNAs and miRNAs were identified respectively. The ceRNA network was then constructed and contained 59 lncRNAs, 23 DEmiRNAs, and 52 DEmRNAs. In total, 5 lncRNAs (HCG22, ADAMTS9-AS1, ADAMTS9-AS2, AC078778.1, and AC112721.1), 2 miRNAs (hsa-mir-145 and hsa-mir-141) and 6 mRNAs (ZEB1, TMEM100, MAP1B, DUSP2, JUN, and AIFM3) were found to be related to OS. Two lncRNAs (ADAMTS9-AS1 and ADAMTS9-AS2) and 4 mRNA (DUSP2, JUN, MAP1B, and TMEM100) were validated using GEPIA. Thirty key hub genes were identified using the ranking method of degree. KEGG analysis demonstrated that the majority of the DEmRNAs were involved in pathways associated with cancer.

**Conclusion:**

Our findings provide an understanding of the important role of lncRNA–related ceRNAs in BUC. Additional experimental and clinical validations are required to support our findings.

## Background

Bladder cancer (BC) is the most common urologic cancer. Approximately 429,800 newly diagnosed cases and 165,100 deaths are recorded worldwide every year [1]. The main pathological bladder cancer types are bladder urothelial carcinoma (BUC), bladder adenocarcinoma and bladder squamous cell carcinoma. The most common type is BUC and accounts for more than 90% of all BCs [2]. Unfortunately, the high recurrence rate is a characteristic of BUC [3, 4]. Currently, the principal treatment strategy for BUC consists of surgery and adjuvant combination chemotherapy. However, chemotherapy resistance reduces the sensitivity of BUC to chemotherapeutic drugs and frequently results in treatment failure resulting in BUC clinical management being a major challenge [5]. In addition, only a limited number of biomarkers are available for diagnosing BUC compared to other cancers. Hence, identifying sensitive and specific BUC biomarkers, as well as therapeutic targets for BUC are critically needed.

Long non-coding RNAs (lncRNAs) are a subtype of ncRNAs with transcript lengths over 200 nucleotides and have recently attracted increased attention [6]. lncRNAs were initially regarded as transcriptional noise without the capacity to encode proteins [7]. However, growing evidence has demonstrated that lncRNAs may play crucial biological roles in a variety of biological processes that are associated with carcinogenesis and cancer metastasis [8]. With regards to bladder cancer, several studies have suggested that lncRNAs may function as oncogenes or tumor suppressors and may affect overall patient survival and mortality [9, 10]. To date, only a few lncRNAs have been verified experimentally, but their roles in regulating gene expression remains to be deciphered.

Considerable efforts have been made to demonstrate how lncRNAs exert their diverse biological functions in human malignant tumors. Rapid progression has been made to elucidate the role of lncRNAs in miRNA function. miRNAs are endogenous single-stranded RNA with lengths between 20 and 25 nucleotides that do not encode proteins. They repress gene expression by complement binding to their target mRNA sequences (i.e. microRNA response element, MRE) [11]. In 2011, Salmena et al. [12] proposed the competing endogenous RNA (ceRNA) hypothesis, which states that mRNAs, transcribed pseudogenes, and lncRNAs could act as natural miRNA “sponges” and inhibit miRNA function by competing with the binding of one or more MREs in complex and comprehensive regulatory networks, leading to pathogenic conditions. The ceRNA regulation theory has been proven to be involved in bladder cancer initiation and progression in several studies [13, 14]. Similarly, lncRNAs acting as ceRNAs has also been reported in other cancers [15–17]. Recently, Kouhsar et al. [18] constructed a ceRNA network related to the staging of Non-Muscle Invasive Bladder Cancer (Ta and T1) derived from public data sources. They identified several biomarkers associated with tumor stage. We hypothesized that lncRNAs may function as ceRNAs during BUC initiation and progression. Understanding how lncRNAs function as ceRNAs will be important in deciphering BUC carcinogenesis.

In the present study, we aimed to decipher the regulatory ceRNA network of lncRNAs–miRNAs–mRNAs in BUC by analyzing gene expression data. This was performed using bioinformatics prediction and correlation analyses. In addition, using clinical trials and survival analyses, we identified potential prognostic genes.

## Materials and methods

### Study cohort

RNA sequence data from 406 BUC patients were retrieved from The Cancer Genome Atlas (TCGA) database (https://cancergenome.nih.gov/) in 2018. LncRNA, miRNA, and mRNAseq data were obtained using the Data Transfer Tool (provided by GDC Apps). Patient clinical information was also downloaded using the Data Transfer Tool. Sequencing data derived from the Illumina HiSeq RNAseq and Illumina HiSeq miRNAseq platforms were publicly available. This study met the publication guidelines stated by TCGA (https://cancergenome.nih.Gov/publications/publicationguidelines). All data used in the study were obtained from TCGA, and hence ethics approval and informed consent were not required.

### Differential expression analysis

The mRNAseq and lncRNAseq data derived from 414 BUC tissue samples and 19 non-tumor adjacent tissue samples were downloaded from TCGA. The BUC miRNAseq data were derived from 418 BUC tissue samples and 19 non-tumor adjacent tissue samples. For tumor and non-tumor group comparison, differentially expressed mRNAs (DEmRNAs), miRNAs (DEmiRNAs) and lncRNAs (DElncRNAs) were identified using the “edgeR” package (http://bioconductor.org/packages/release/bioc/html/edgeR.html) with a cut-off criteria of |log2 (fold change [FC])| > 2.0 and adjusted *P* value < 0.01 [19]. Differentially expressed lncRNAs (DElncRNAs) were defined and annotated using the Encyclopedia of DNA Elements (ENCODE), which included 15,877 human lncRNAs. All *P*-values used the False discovery rate (FDR) to correct for statistical significance of multiple testing (Benjamini–Hochberg method) [20]. FDR significance level was set at 0.05.

### KEGG enrichment analysis of DEmRNAs

KEGG enrichment analysis was performed using the “clusterProfiler” package in R software based on the retrieved DEmRNAs and visualized using the Cytoscape v 3.5.1 software.

### Protein–protein interaction (PPI) network

To understand the interactions of the DEmRNAs, we constructed a PPI network using the Search Tool for the Retrieval of Interacting Genes (STRING, http://string.embl.de/). Combined scores greater than 0.4 were considered statistically significant. The PPI network was visualized using the Cytoscape v 3.5.1 software. Subsequently, the top 30 mRNAs were identified using the ranking method of degree. In addition, gene ontology (GO) enrichment analysis was performed using STRING to functionally annotate the DEmRNAs in BUC.

### ceRNA network

In order to investigate the role of the differentially expressed RNAs in the ceRNA network, a dysregulated lncRNA–miRNA–mRNA ceRNA network was constructed and visualized using the Cytoscape v 3.5.1 software. LncRNA–miRNA interactions were predicted using miRcode (http://www.mircode.org/). miRNA–targeted mRNAs were predicted using the miRTarBase (http://mirtarbase.mbc.nctu.edu.tw/), miRDB (http://www.mirdb.org/), and TargetScan (http://www.targetscan.org/vert_71/). Each regulatory pair of miRNAs and mRNAs were verified using quantitative real-time PCR, western blot, reporter assays, microarrays, and next-generation sequencing data derived from miRTarBase.

### Independent prognostic factors for overall survival (OS)

Independent prognostic differentially expressed RNAs for OS were identified by univariate and multivariate cox regression analyses. The risk score (RS) was estimated using the following formula:

$${\text{RS}} = \sum\limits_{i = 1}^{n} {{\text{Exp}}(i)R(i)} ,$$

where Exp(i) denotes the expression value for RNA; n represents the number of RNA modules, and R(i) denotes the estimated regression coefficient of RNA. If the RS for a given sample was greater than the mean RS of all samples, the latter was regarded as a high-risk sample, otherwise, it was regarded as a low-risk sample. Kaplan–Meier method was used to evaluate the survival curves in the high- and low-risk groups. Additionally, the sensitivity and specificity were accessed using the receiver operating characteristic (ROC) curves and area under the ROC curves (AUC values).

Survival curves were plotted using the “survival” package in R for the independent prognostic RNAs that were identified. Long-rank test was used to evaluate statistical significance and P < 0.05 was considered statistically significant.

### Validating the prognostic value of lncRNAs and mRNAs in the ceRNA network using GEPIA

Independent prognostic lncRNAs and mRNAs were validated using Gene Expression Profiling Interactive Analysis (GEPIA,http://gepia.cancerpku.cn/index.htm), which was based on RNA sequencing data from 9736 tumors and 8587 normal samples in the Cancer Genome Atlas (TCGA) and Genotype-Tissue Expression (GTEx) dataset project [21]. In addition, the correlation between lncRNAs and mRNAs was confirmed using the Pearson correlation statistic. The correlation of two RNAs was considered significant when R was greater than 0.4 and the P-value was less than 0.05.

## Results

### Identifying DElncRNAs, DEmRNAs, and DEmiRNAs

A total of 666 DElncRNAs, 1819 DEmRNAs and 157 DEmiRNAs were identified with |log2FC| > 2.0 and adjusted P-value < 0.01 using the “edgeR” package. Heat maps with complete linkage clustering of differentially expressed RNAs were performed using the “gplots” package (Additional file 1: Figure S1A–C). The results identified; 420 (63.1%) up-regulated and 246 (36.9%) down-regulated DElncRNAs, 1030 (56.6%) up-regulated and 789 (43.4%) down-regulated DEmRNAs, and 131 (83.4%) up-regulated and 26 (16.6%) down-regulated DEmiRNAs.

### Pathway enrichment analysis of DEmRNAs

To investigate the mechanisms associated with BUC tumorigenesis, 1819 DEmRNAs were used for KEGG enrichment analysis. The threshold was set at *P *< 0.01. The top 15 significantly enriched pathways are presented in Table 1. The results showed that the majority of DEmRNAs were enriched for “neuroactive ligand–receptor interaction”, “viral carcinogenesis”, “protein digestion and absorption”, “ECM–receptor interaction”, and “cAMP signaling pathway”. In addition, a network of pathways and DEmRNAs was constructed based on the above KEGG analysis and visualized using Cytoscape v 3.5.1 (Additional file 2: Figure S2). Interestingly, several DEmRNAs were related to more than one pathway, such as SLC8A1, CAMK2B, and CACNA2D1. CAMK2B was enriched for Circadian entrainment, calcium signaling pathway, adrenergic signaling in cardiomyocytes, cAMP signaling pathway, oxytocin signaling pathway, and insulin secretion. These DEmRNAs may be associated with BUC carcinogenesis and progression.

**Table 1:** Top 15 significantly enriched pathways derived from the DEmRNAs

ID	Description	P value	P adjust	q value	Gene ID	Count
hsa05322	Systemic lupus erythematosus	5.1E−20	1.47E−17	1.23E−17	ELANE/C7/HIST3H2A/CTSG/HIST1H2AD/HIST1H3D/HIST1H2BD/ HIST2H2BF/HIST1H3G/HIST1H2AE/HIST1H2BJ/HIST3H2BB/ HIST1H2BO/HIST1H4I/HIST1H2BG/HIST1H2BH/HIST1H2AG/ HIST1H2BE/HIST1H2BF/HIST1H2BC/HIST1H2BK/HIST1H2AI/ HIST1H3B/HIST1H3H/HIST1H4E/HIST2H4A/HIST1H2AM/ HIST1H2AH/HIST1H3C/HIST1H2BM/HIST1H4D/HIST1H2AL/ HIST1H2BN/HIST1H2BI/HIST1H3F/HIST2H3D/HIST1H4C/ HIST1H4B/HIST1H3I/HIST2H2AB/HIST1H3J/HIST1H2BL/ HIST1H2AB/HIST1H3A/HIST1H4A/HIST1H4H/FCGR3A/ HIST1H2BB/HIST1H2AJ/H2BFM/HIST1H4F/HIST1H4L	52
hsa05034	Alcoholism	3.48E−18	5.02E−16	4.2E−16	FOSB/GNAO1/GNG7/ADCY5/MAOB/HIST3H2A/HIST1H2AD/ HIST1H3D/HIST1H2BD/HIST2H2BF/HIST1H3G/HIST1H2AE/ HIST1H2BJ/GRIN2D/HIST3H2BB/HIST1H2BO/HIST1H4I/ HIST1H2BG/HIST1H2BH/GRIN3B/HIST1H2AG/HIST1H2BE/ HIST1H2BF/HIST1H2BC/HIST1H2BK/HIST1H2AI/HIST1H3B/ HIST1H3H/HIST1H4E/HIST2H4A/HIST1H2AM/HIST1H2AH/ HIST1H3C/HIST1H2BM/HIST1H4D/HIST1H2AL/HIST1H2BN/ HIST1H2BI/HIST1H3F/HIST2H3D/HIST1H4C/HIST1H4B/ HIST1H3I/HIST2H2AB/HIST1H3J/HIST1H2BL/HIST1H2AB/ GRIN1/HIST1H3A/HIST1H4A/HIST1H4H/HIST1H2BB/ HIST1H2AJ/GNG4/GNG13/H2BFM/HIST1H4F/TH/HIST1H4L	59
hsa04080	Neuroactive ligand–receptor interaction	9.5E−10	9.12E−08	7.63E−08	GLP2R/TACR2/P2RX1/P2RY14/VIPR2/TACR3/PTGFR/CHRM2/ ADRB3/AGTR1/CHRM3/LEPR/ADCYAP1R1/PTH1R/BDKRB1/ BDKRB2/ADRA1D/GHR/HTR1B/CTSG/TACR1/GRIN2D/ CHRNA1/GRIN3B/PTGER3/GRIK3/HTR2A/GABRG1/SSTR1/ S1PR5/GALR1/LHB/PRSS2/PRSS1/GRIN1/GRM4/MTNR1B/ GABRA4/KISS1R/OXTR/CHRNG/CHRNB2/PRLHR/CHRND/ CHRNA9/GRM3/GALR2/CHRNA6/GPR83/GLP1R/GLRA3/ SCTR/F2/DRD5/GABRR3/HTR2C/RXFP3/GHRHR/GABBR2/PRL	60
hsa05410	Hypertrophic cardiomyopathy (HCM)	1.34E−09	9.68E−08	8.1E−08	TPM1/DMD/ITGA8/CACNB2/ACTC1/CACNA1C/TPM2/ITGA7/ SGCA/RYR2/SLC8A1/LAMA2/DES/SGCD/SGCG/ITGA5/ITGA9/ ITGA1/TNNT2/IL6/CACNB4/PRKAA2/CACNA2D1/ITGB3/ MYL3/TNNI3/CACNG4/CACNG1	28
hsa05414	Dilated cardiomyopathy (DCM)	2.31E−09	1.33E−07	1.11E−07	TPM1/DMD/ITGA8/CACNB2/ACTC1/CACNA1C/TPM2/PLN/ ITGA7/SGCA/RYR2/SLC8A1/LAMA2/DES/ADCY5/SGCD/ SGCG/ITGA5/ITGA9/ITGA1/TNNT2/CACNB4/CACNA2D1/ ITGB3/MYL3/ADCY2/TNNI3/CACNG4/CACNG1	29
hsa04020	Calcium signaling pathway	6.17E−08	2.96E−06	2.48E−06	PLCD4/MYLK/TACR2/ITPKB/RYR3/CACNA1H/PDE1C/P2RX1/ ITPR1/GNAL/CACNA1C/PLN/PDE1A/CAMK2A/TACR3/RYR2/ PTGFR/SLC8A1/PLCB4/CHRM2/ADRB3/AGTR1/CHRM3/ BDKRB1/BDKRB2/ADRA1D/PRKCB/TACR1/SLC8A3/GRIN2D/ ADCY2/PTGER3/SLC8A2/HTR2A/GRIN1/OXTR/CAMK2B/ CACNA1E/MYLK4/DRD5/CACNA1B/HTR2C	42
hsa05412	Arrhythmogenic right ventricular cardiomyopathy (ARVC)	5.72E−07	2.35E−05	1.97E−05	DMD/ITGA8/ACTN2/CACNB2/CACNA1C/ITGA7/SGCA/RYR2/ SLC8A1/LAMA2/DES/SGCD/SGCG/ITGA5/ITGA9/ITGA1/ CACNB4/CACNA2D1/ITGB3/CTNNA3/CACNG4/CACNG1	22
hsa04713	Circadian entrainment	2.72E−06	8.74E−05	7.32E−05	RYR3/CACNA1H/PER2/ITPR1/FOS/CACNA1C/GNAO1/CAMK2A/ GNG7/PRKG1/RYR2/PLCB4/ADCY5/PER1/ADCYAP1R1/PRKCB/ KCNJ3/GRIN2D/ADCY2/GRIN1/MTNR1B/CAMK2B/KCNJ6/ GNG4/GNG13	25
hsa04974	Protein digestion and absorption	2.73E−06	8.74E−05	7.32E−05	ATP1A2/SLC8A1/COL14A1/ATP1B2/XPNPEP2/COL10A1/CPA3/ COL21A1/SLC8A3/COL11A1/ELN/COL4A4/COL4A6/SLC8A2/ COL7A1/COL2A1/COL6A5/PRSS2/SLC15A1/PRSS1/MEP1A/ ATP1A3/CPB2/SLC6A19	24
hsa04911	Insulin secretion	3.35E−06	9.66E−05	8.09E−05	KCNMA1/KCNMB1/ATP1A2/CACNA1C/CAMK2A/RYR2/PLCB4/ ADCY5/ATP1B2/CHRM3/ADCYAP1R1/ADCYAP1/KCNN2/ PRKCB/ADCY2/ABCC8/PDX1/CAMK2B/ATP1A3/GLP1R/INS/ SLC2A2/GIP	23
hsa05203	Viral carcinogenesis	6.19E−06	0.000162	0.000136	EGR3/GSN/JUN/CDK1/EGR2/CDC20/CCNE1/HIST1H2BD/ HIST2H2BF/HIST1H2BJ/CCNE2/HIST3H2BB/HIST1H2BO/ HIST1H4I/HIST1H2BG/HIST1H2BH/HIST1H2BE/HIST1H2BF/ HIST1H2BC/HIST1H2BK/HIST1H4E/GTF2A1L/HIST2H4A/ CCR3/CDKN2A/HIST1H2BM/HIST1H4D/HIST1H2BN/ HIST1H2BI/HIST1H4C/ATP6V0D2/HIST1H4B/HIST1H2BL/ HIST1H4A/HIST1H4H/HIST1H2BB/H2BFM/HIST1H4F/ HPN/HIST1H4L	40
hsa04512	ECM–receptor interaction	2.24E−05	0.000538	0.00045	TNXB/ITGA8/ITGA7/THBS1/LAMA2/ITGA5/ITGA9/ITGA1/ LAMC3/ITGB3/HMMR/COL4A4/COL4A6/SPP1/IBSP/ COL2A1/COL6A5/LAMC2/GP6/COMP/TNN	21
hsa04921	Oxytocin signaling pathway	0.000104	0.00231	0.001933	PPP1R12B/MYLK/RYR3/MYL9/ITPR1/RCAN1/FOS/ CACNB2/CACNA1C/GNAO1/CAMK2A/RGS2/RYR2/ PLCB4/JUN/ADCY5/CACNB4/PRKAA2/CACNA2D1/ PRKCB/PTGS2/KCNJ3/ADCY2/OXTR/CACNG4/ CAMK2B/KCNJ6/MYLK4/KCNJ4/CACNG1	30
hsa04024	cAMP signaling pathway	0.000131	0.002696	0.002257	MYL9/FOS/ATP1A2/PDE4D/CACNA1C/PLN/ CAMK2A/FXYD1/VIPR2/CNGA3/RYR2/JUN/CHRM2/ ADCY5/ATP1B2/ADCYAP1R1/RAC3/HTR1B/HHIP/ GRIN2D/ADCY2/GRIN3B/TNNI3/PTGER3/CNGB1/ SSTR1/CNGB3/AMH/GRIN1/OXTR/CAMK2B/ SUCNR1/ATP1A3/GLP1R/DRD5/GABBR2	36
hsa04261	Adrenergic signaling in cardiomyocytes	0.000229	0.004393	0.003677	TPM1/CACNB2/ACTC1/ATP1A2/CACNA1C/TPM2/ PLN/CAMK2A/RYR2/SLC8A1/PLCB4/ADCY5/ ATP1B2/TNNT2/AGTR1/SCN7A/CACNB4/SCN4B/ PPP1R1A/ADRA1D/CACNA2D1/MYL3/ADCY2/ TNNI3/CACNG4/CAMK2B/ATP1A3/CACNG1	28

### Functional enrichment analysis of DEmRNAs

**Fig. 1:**
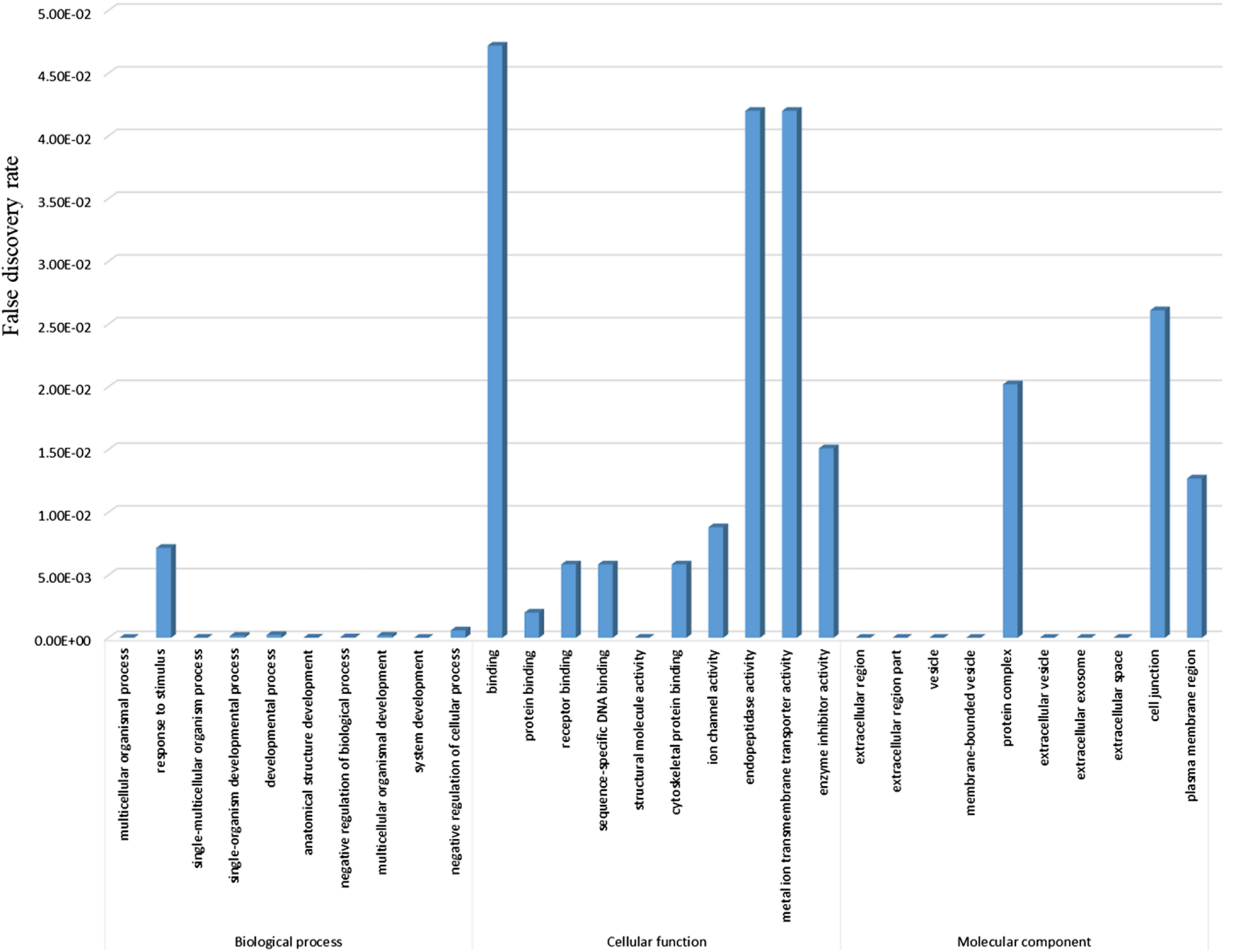
GO analysis of the top 600 significant DEmRNAs [300 significantly up-regulated and 300 significantly down-regulated DEmRNAs based on log fold change (log FC). Horizontal axis: gene ontology (GO) annotation, Vertical axis: false discovery rate (FDR)]

Functional enrichment of the top 600 DEmRNAs were analyzed using STRING (300 significantly up-regulated and 300 significantly down-regulated DEmRNAs based on log fold change (log FC)). The GO function for the various genes were divided into biological processes (BP), cellular function (CF) and molecular component (MC). The GO results are presented in Fig. 1. The results from the GO functional enrichment analysis suggested that DEmRNAs were significantly enriched in multicellular organisms (ontology: BP), binding (ontology: CF) and extracellular region (MC). These results demonstrated that the enriched DEmRNAs were associated with BUC proliferation and migration.

### Protein–protein interaction (PPI) network

**Fig. 2:**
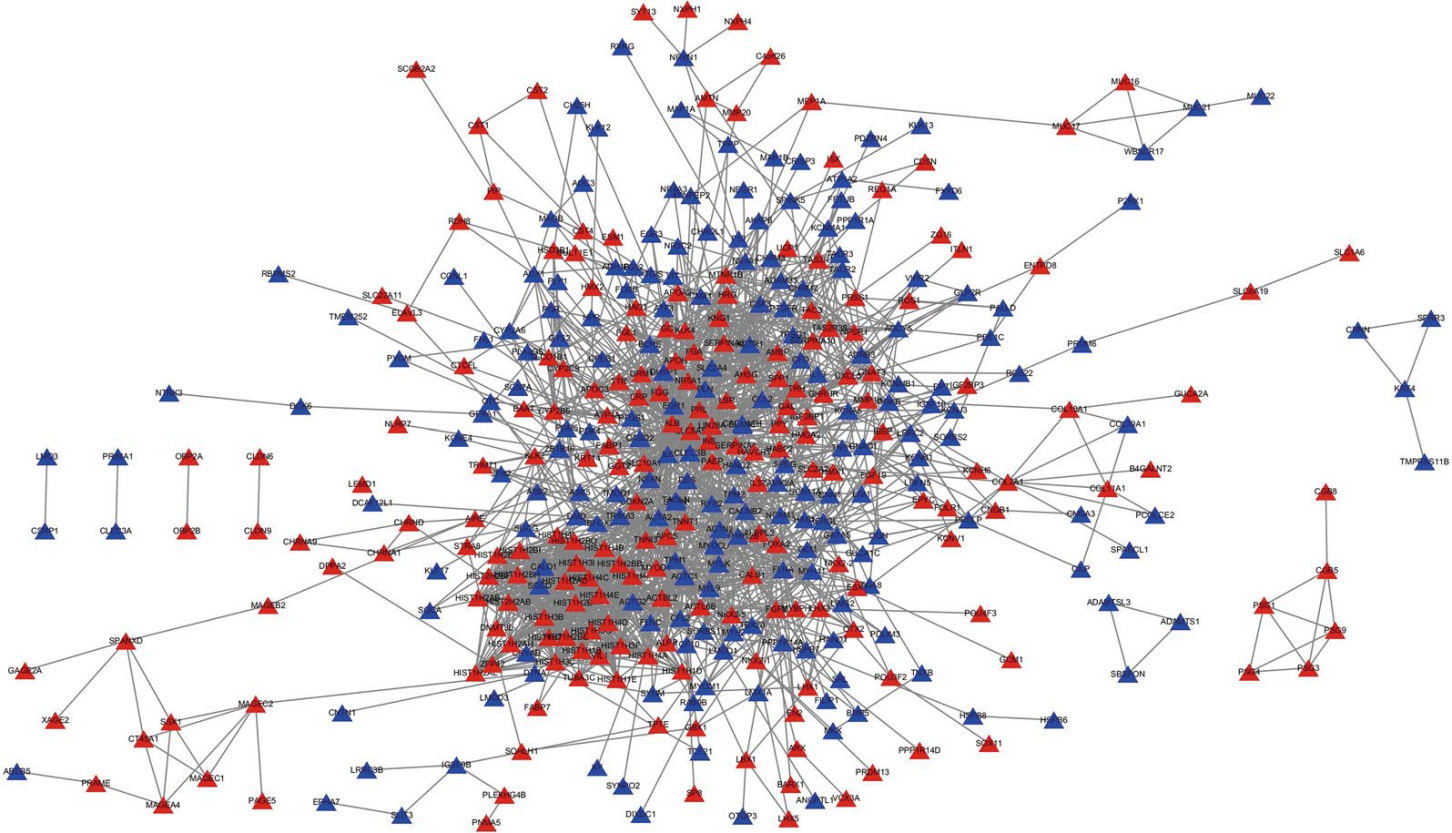
PPI networks of the DEmRNAs [300 significantly up-regulated and 300 significantly down-regulated DEmRNAs based on log fold change (log FC)] constructed for BUC. Each triangle corresponds to a protein-coding gene (mRNA). Each edge represents the possible associations between genes. Red triangles represent the up-regulated DEmRNAs; blue tringles represent down-regulated DEmRNAs

The interrelationship between the top 600 significant DEmRNAs (300 significantly up-regulated and 300 significantly down-regulated DEmRNAs based on log fold change (log FC)) were retrieved from the STRING database to construct the PPI network (Fig. 2). The PPI network consisted of 418 nodes and 1937 edges. The nodes denoted DEmRNAs, while the edges denoted interactions among the DEmRNAs. Additionally, the top 30 mRNAs in the PPI network were analyzed using the ranking method of degree (Additional file 3: Figure S3). KEGG pathway enrichment analysis for the 30 mRNAs identified with a high degree was performed using the “clusterProfiler” package in the R software with a *P *< 0.05 as the cut-off criteria. The results demonstrated that these mRNAs were enriched for pathways related to “Systemic lupus erythematosus”, “Alcoholism”, “viral carcinogenesis” and “transcriptional dysregulation in cancer” (Additional file 4: Figure S4A). Furthermore, we identified 21 (HIST2H2BF, HIST1H2BO, HIST1H2BH, HIST1H2BE, HIST1H2BF, HIST1H4E, HIST1H2BM, HIST1H4D, HIST1H2BI, HIST1H4C, HIST1H4B, HIST1H4A, HIST1H2BB, HIST1H4F, HIST1H4L, IL6, HIST1H3G, HIST1H3B, HIST1H3C, HIST1H3F and HIST1H3I) of these 30 DEmRNAs that were enriched for cancer-related pathways: “viral carcinogenesis” or “transcriptional dysregulation in cancer”. These 21 DEmRNAs were classified as hub genes. In addition, a network linking the pathways and mRNAs were constructed and visualized using Cytoscape v 3.5.1 (Additional file 4: Figure S4B).

### Construction of the ceRNA network for BUC

**Fig. 3:**
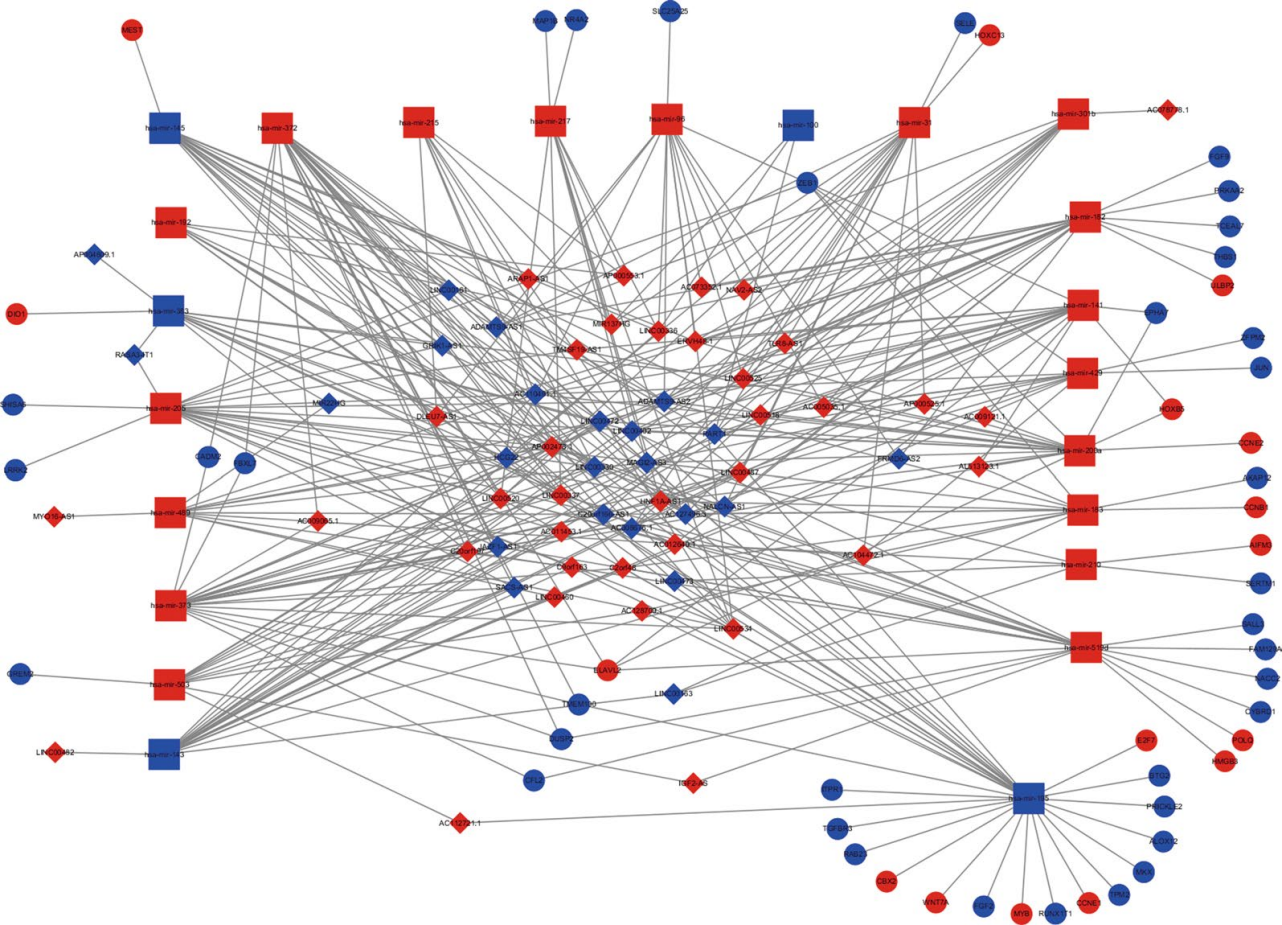
CeRNA network built on BUC. The red nodes represent the upregulated, while the blue nodes represent the downregulated mRNAs. Ellipse nodes denote DEmRNAs; Diamond nodes denote DElncRNAs; rectangle nodes denote DEmiRNAs. Gray edges denote lncRNA–miRNA–mRNA interactions

To better understand the role of the identified differentially expressed RNAs in BUC, a dysregulated ceRNA network based on DElncRNA–DEmiRNA–DEmRNA interactions was constructed using Cytoscape v 3.5.1 (Fig. 3). The regulatory relationship between DEmiRNAs and DElncRNAs pairs were retrieved from miRcode (http://www.mircode.org/). All the DEmiRNAs and DElncRNAs involved in the ceRNA network meet the cut-off criteria (|log2FC| > 2.0 and adjusted P-value < 0.01) mentioned above. We found 259 lncRNA–miRNA interaction pairs containing 59 lncRNAs and 23 DEmiRNAs from the miRcode database. Subsequently, we searched for mRNAs that were targeted by the 23 DEmiRNAs using miRTarBase (http://mirtarbase.mbc.nctu.edu.tw/), miRDB (http://www.mirdb.org/) and TargetScan (http://www.targetscan.org/vert_71/). All the DEmRNAs meet the cut-off criteria mentioned above (|log2FC| > 2.0 and adjusted P-value < 0.01). Finally, 52 DEmRNAs including 70 miRNA–mRNA interaction pairs were included in our ceRNA network.

### Independent prognostic factors for overall survival

**Fig. 4:**
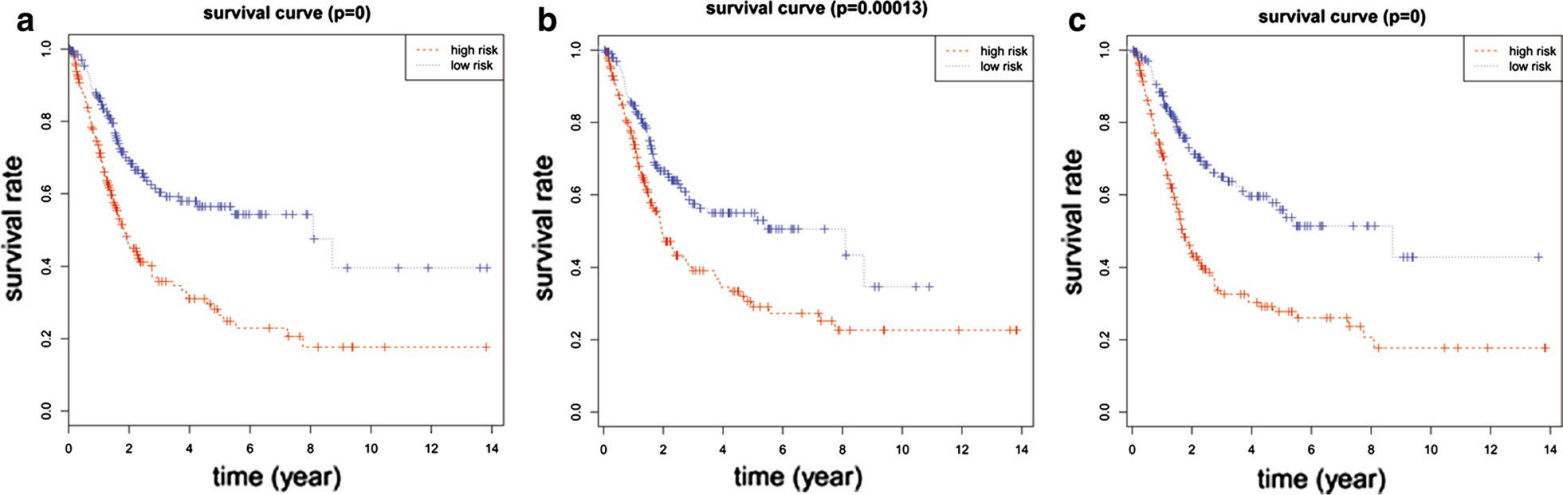
Survival of high versus low risk differentially expressed RNAs associated with independent prognostic factors (**a** for DElncRNAs, **b** for DEmiRNAs and **c** for DEmRNAs). DElncRNA, differentially expressed long noncoding RNA; DEmiRNA, differentially expressed microRNA; DEmRNA, differentially expressed messenger RNA

**Fig. 5:**
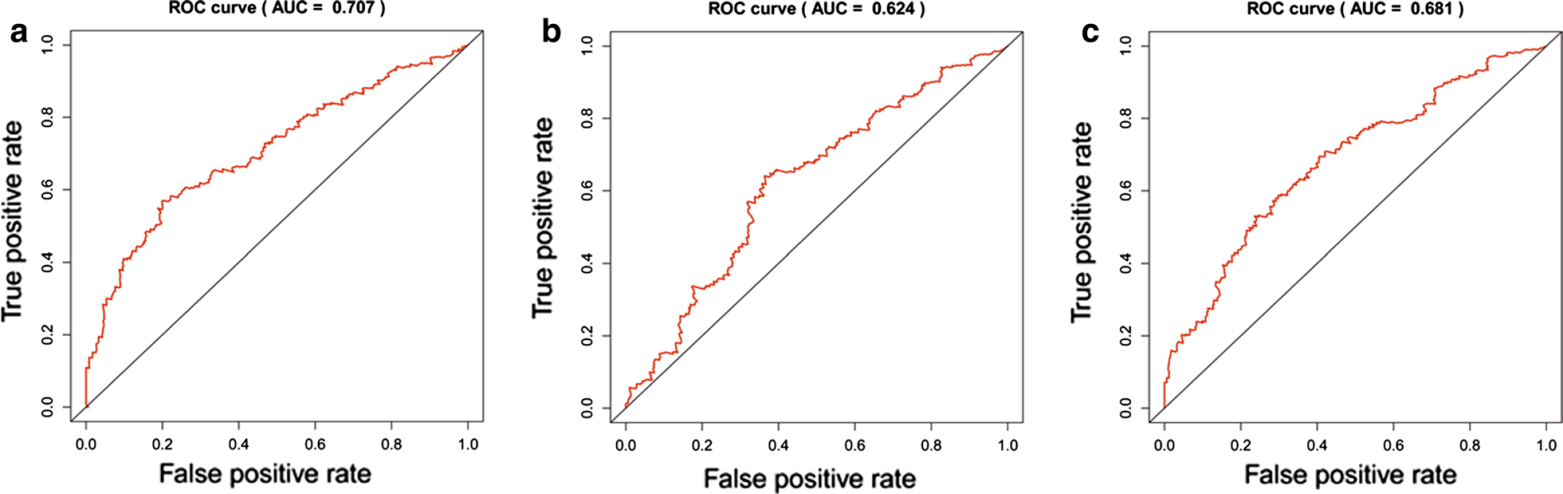
Receiver operating characteristic (ROC) curve analysis and area under the curve (AUC) value for the ROC curve indicating the sensitivity and specificity of the independent prognostic differentially expressed RNAs (including DElncRNA, DEmiRNAs, and DEmRNAs) for survival prediction (**a** for DElncRNA, **b** for DEmiRNAs and **c** for DEmRNAs)

59 DElncRNAs, 23 DEmiRNAs and 52 DEmRNAs in the ceRNA network were included in univariate cox regression analysis. 17 DElncRNAs, 6 DEmiRNAs and 19 DEmRNAs (P < 0.05 in univariate cox regression analysis) were identified for multivariable cox regression analysis. Multivariable analysis suggested that 7 lncRNAs (HCG22, ADAMTS9-AS1, ADAMTS9-AS2, AC078778.1, AC112721.1, LINC00525 and NAV2-AS2), 3 DEmiRNAs (hsa-mir-145, hsa-mir-141 and hsa-mir-373), and 7 DEmRNAs (ZEB1, TMEM100, MAP1B, DUSP2, JUN, AIFM3 and MEST) were closely related to OS in BUC patients (*P *< 0.05) (Table 2 and heat maps in Additional file 5: Figure S5A–C). Based on risk scores (RS) of the independent prognostic RNAs identified above, patients included in the present study were assigned into high- and low-risk groups. The low-risk group had a significantly better prognosis compared to the high-risk group (Fig. 4a for lncRNA, B for miRNA and C for mRNA). The results showed that the 5-year OS for the low-risk group was 56.4%, 55.0%, and 55.8% respectively, while it was 26.5%, 29.1% and 27.8% for the high-risk group, respectively. To determine the prognostic power of the 7 lncRNAs, 3 DEmiRNAs, and 7 DEmRNAs identified above, time-dependent receiver operating characteristic (ROC) curve analysis was performed and the area under the curve (AUC values) was estimated. AUC values for the independent prognostic DElncRNAs, DEmiRNAs, and DEmRNAs were 0.707, 0.624 and 0.681, respectively (Fig. 5, a for lncRNA, b for miRNA, C for mRNA), indicating good specificity and sensitivity.

**Table 2:** Univariate and multivariable Cox regression analysis of RNAs involved in the ceRNA network

RNA	Univariate analysis			Multivariate analysis	
	HR	z	P value	Coef	P-value
DElncRNA					
HCG22	1.114809	2.286046	0.022252	0.0832	0.12686
ADAMTS9-AS1	1.113288	3.316744	0.000911	0.1399	0.01446
ADAMTS9-AS2	1.109552	2.379209	0.01735	− 0.2156	0.00652
AC078778.1	0.701409	− 4.44072	8.97E−06	− 0.3555	0.00016
AC112721.1	1.149849	3.455774	0.000549	0.1228	0.01101
LINC00525	0.922187	− 2.09449	0.036217	− 0.0950	0.01627
NAV2-AS2	1.22102	3.352368	0.000801	0.1956	0.00127
DEmiRNA					
hsa-mir-145	1.147843	2.801917	0.00508	0.1150	0.025
hsa-mir-141	0.889523	− 2.78119	0.005416	− 0.0851	0.071
hsa-mir-373	1.117585	2.206949	0.027318	0.1211	0.019
DEmRNA					
ZEB1	1.145221	2.29892	0.021509	− 0.2108	0.0289
TMEM100	1.109099	2.852211	0.004342	0.0773	0.1204
MAP1B	1.229249	4.381164	1.18E−05	0.1810	0.0126
DUSP2	0.874843	− 2.91232	0.003588	− 0.1150	0.0257
JUN	1.209572	2.808707	0.004974	0.1721	0.0204
AIFM3	0.853472	− 3.5464	0.000391	− 0.1215	0.0068
MEST	1.110213	2.451933	0.014209	0.0953	0.0251

HR > 1, lncRNA was negatively associated with OS

HR < 1, lncRNA was positively associated with OS

*HR* hazard rate, *coef* regression coefficient, *HR > 1* differentially expressed RNA was negatively associated with OS, *HR < 1* differentially expressed RNA was positively associated with OS

**Fig. 6:**
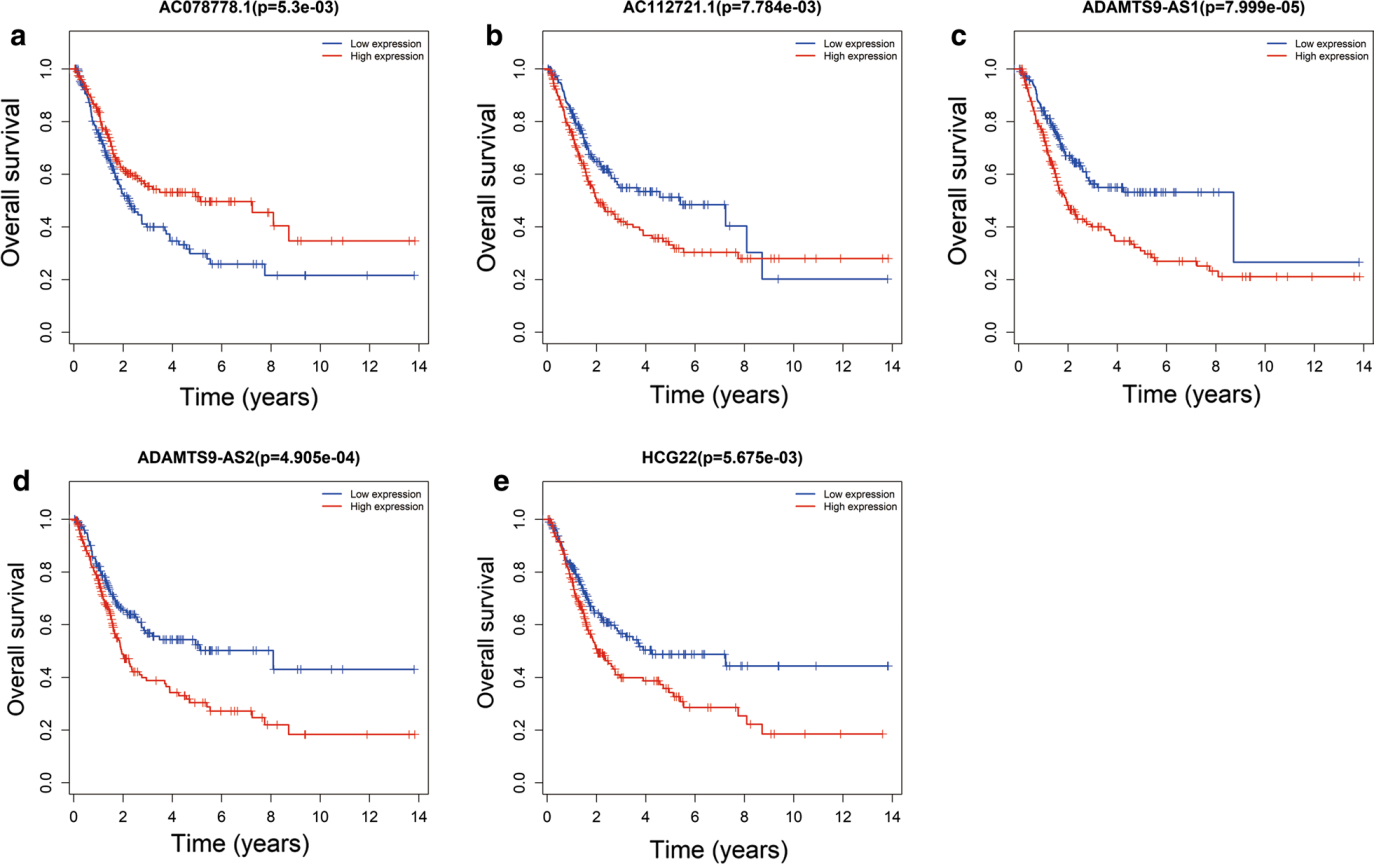
Kaplan–Meier survival curves for 5 DElncRNAs as independent prognostic factors associated with overall survival in BUC. (Five DElncRNA are presented (**a** for AC078778.1, **b** for AC112721.1, **c** for ADAMTS9-AS1, **d** for ADAMTS9-AS2 and **e** for HCG22) (P < 0.05). Horizontal axis: overall survival time: years, Vertical axis: overall survival

**Fig. 7:**
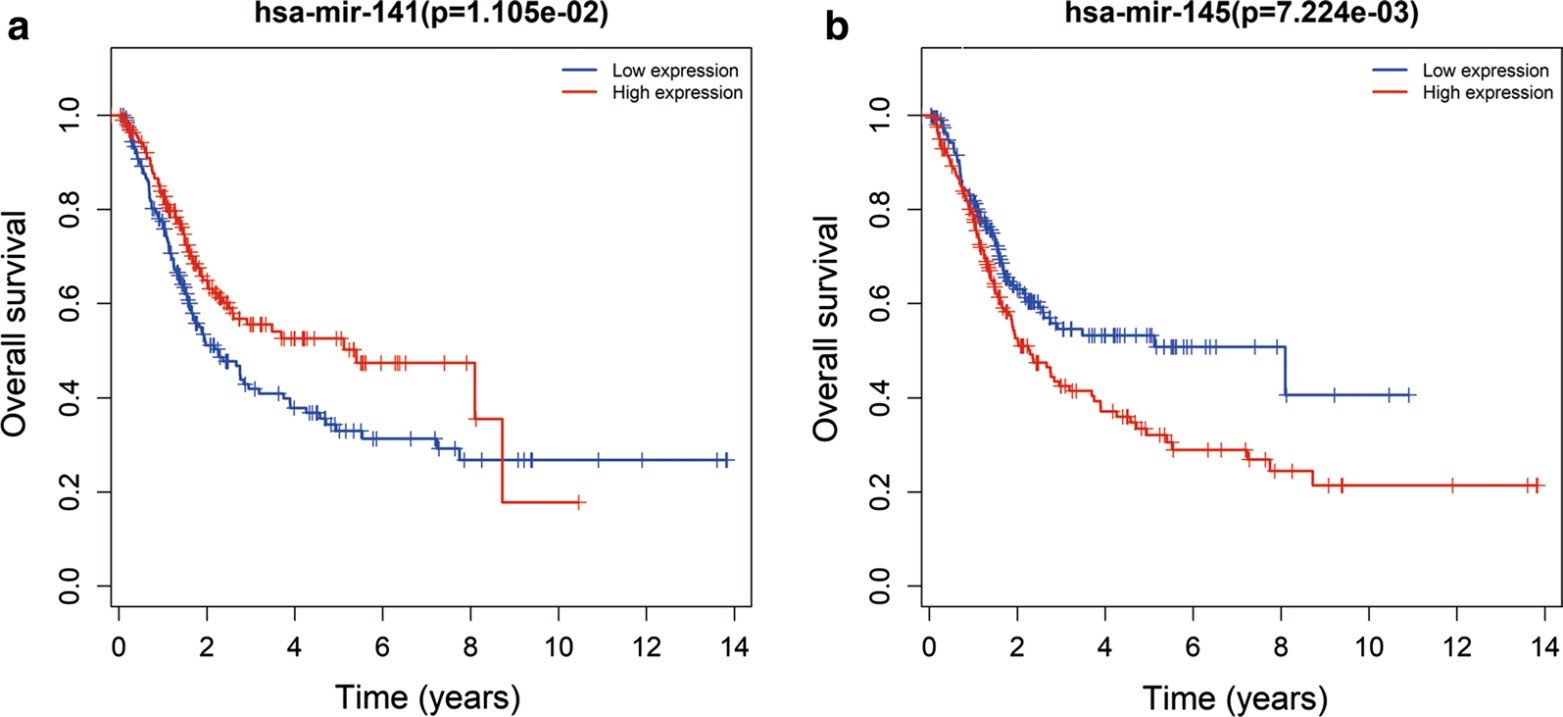
Kaplan–Meier survival curves for two DEmiRNAs that were independent prognostic factors associated with overall survival in BUC. (Two DEmiRNA were selected based on statistical significance (**a** for hsa-mir-141 and **b** for hsa-mir-145) (P < 0.05). Horizontal axis: overall survival time: years, Vertical axis: overall survival

**Fig. 8:**
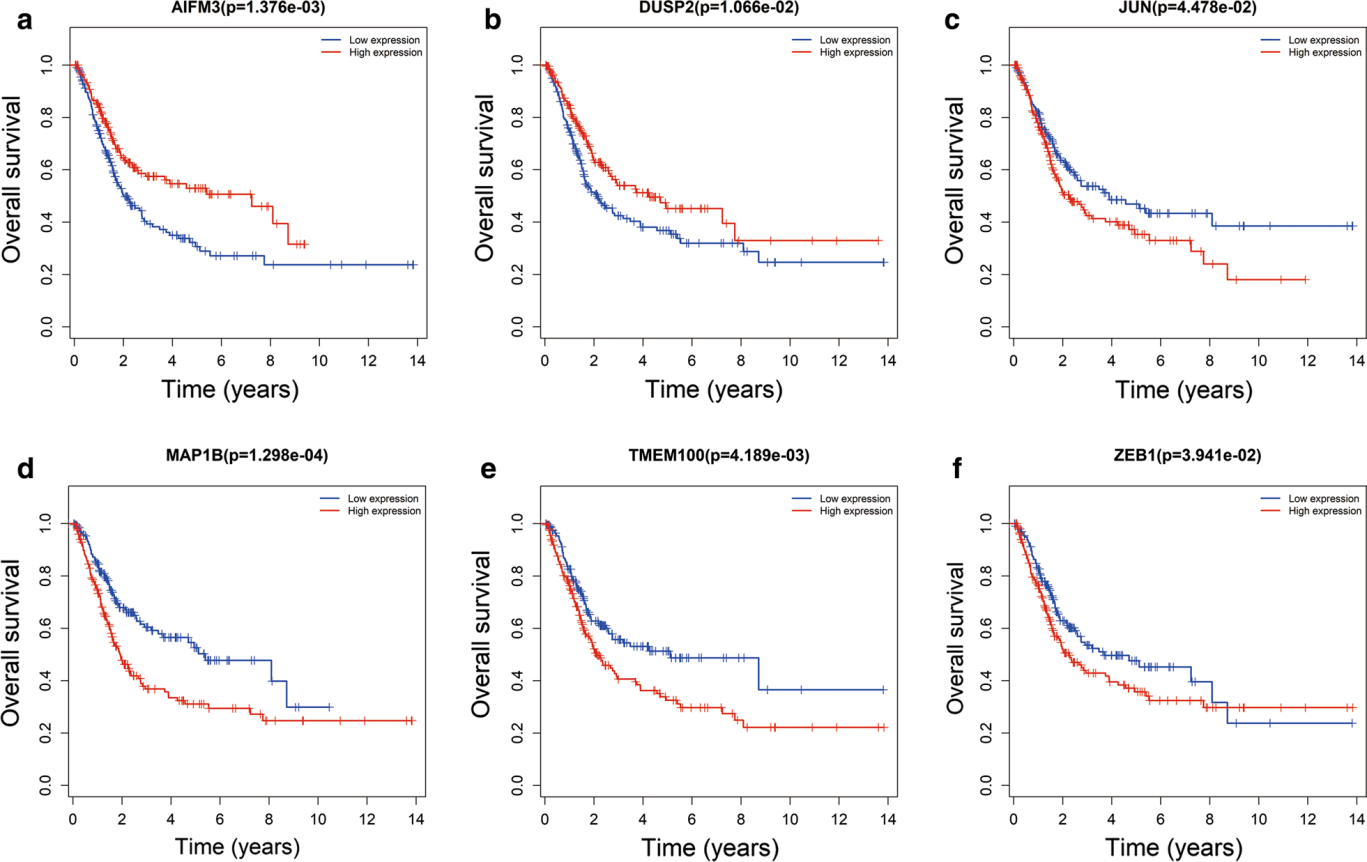
Kaplan–Meier survival curves for the six DEmRNAs independent prognostic factors associated with overall survival in BUC. (Six DEmiRNA were selected based on statistical significance (**a** for AIFM3, **b** for DUSP2, **c** for JUN, **d** for MAP1B, **e** for TMEM100 and **f** for ZEB1) (P < 0.05). Horizontal axis: overall survival time: years, Vertical axis: overall survival

Kaplan–Meier curve analysis was performed to determine the OS for the independent prognostic RNAs. One patient was lost during follow-up and was excluded from the survival analysis. Five DElncRNAs were significantly related to OS, of which, four DElncRNAs (HCG22, ADAMTS9-AS1, ADAMTS9-AS2, and AC112721.1) were negatively related to OS (Fig. 6b–e), while AC078778.1 was positively related to OS (log-rank P < 0.05) (Fig. 6a). In addition, Kaplan–Meier curve analysis for the three DEmiRNAs and seven DEmRNAs showed that two DEmiRNAs (hsa-mir-141 and hsa-mir-145) and 6 DEmRNAs (ZEB1, TMEM100, MAP1B, DUSP2, JUN, and AIFM3) were significantly related to OS (log-rank P < 0.05) (Figs. 7a, b, 8a–f).

### Prognostic value of lncRNAs and mRNAs in the ceRNA network analyzed using GEPIA

**Fig. 9:**
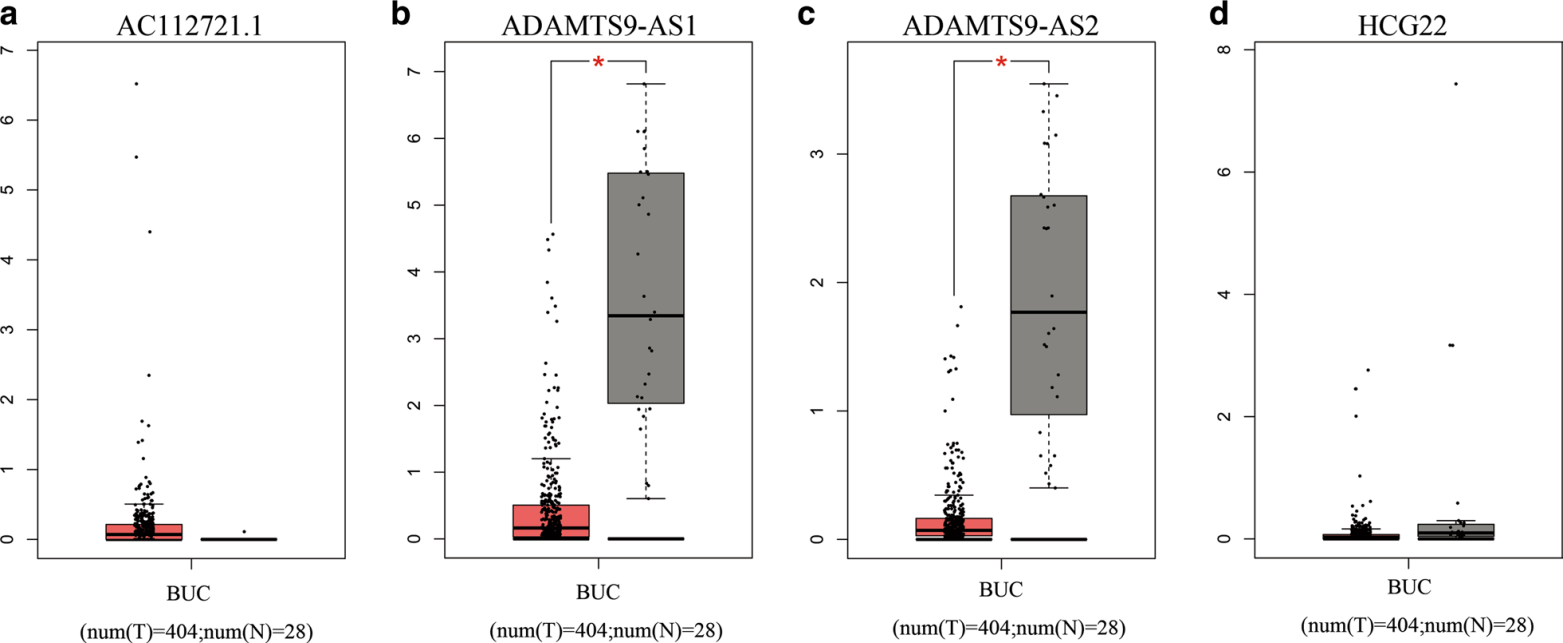
Differential expression of the four-independent prognostic lncRNAs (**a** for AC112721.1, **b** for ADAMTS9-AS1, **c** for ADAMTS9-AS2, and **d** for HCG22) in human BUC versus normal bladder controls analyzed using GEPIA. The red and gray boxes represent BUC and normal tissues respectively. (BUC, bladder urothelial carcinoma. *num* number, *T* tumor, *N* normal. *P < 0.05 and was considered to be statistically significant.)

**Fig. 10:**
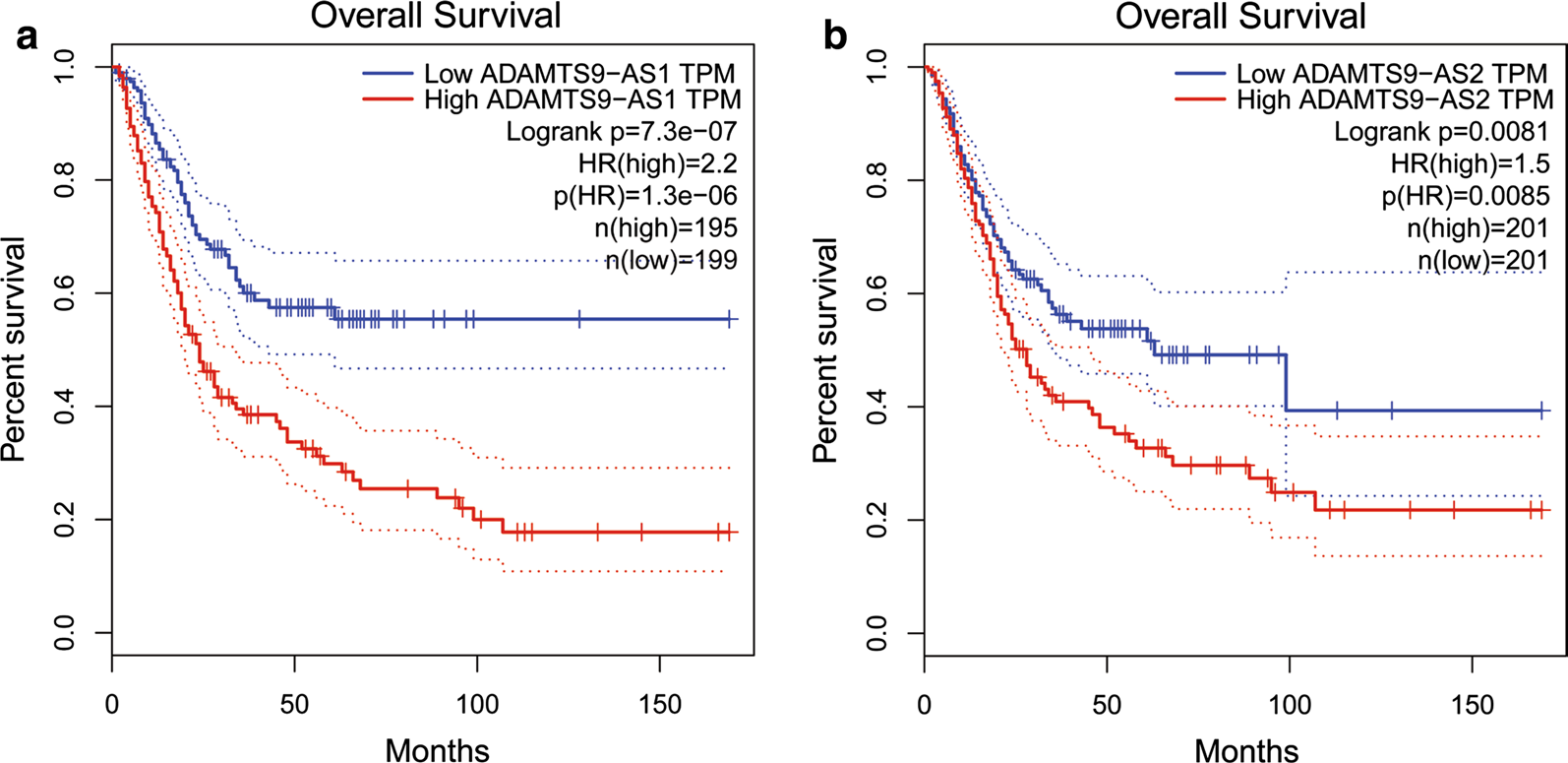
Survival analysis of two independent prognostic lncRNAs (**a** for ADAMTS9-AS1 and **b** for ADAMTS9-AS2) in BUC patients analyzed using GEPIA. The median expression of ADAMTS9-AS1 and ADAMTS9-AS2 was set as the threshold for demarcating high- and low-expression cohorts. Log Rank was used with P < 0.05 considered as statistically significant. *TPM* transcripts per million. Horizontal axis: overall survival time: months, Vertical axis: survival

GEPIA was used to validate the expression levels and prognostic value of the 5 independent lncRNAs. The expression levels of HCG22, ADAMTS9-AS1 and ADAMTS9-AS2 were negative or nearly negative, while AC112721.1 expression levels were positive in BUC tissues. These findings were concordant with previous results using TCGA analysis. However, only ADAMTS9-AS1 and ADAMTS9-AS2 expression levels were down-regulated with statistical significance in BUC. The expression levels of these 4 lncRNAs are shown in Fig. 9. However, AC078778.1 was not found in GEPIA, while the overall survival of the 2 lncRNAs, ADAMTS9-AS1 and ADAMTS9-AS2, were estimated using GEPIA. The results are shown in Fig. 10 and were similar to our previous results. Lower expression of ADAMTS9-AS1 and ADAMTS9-AS2 were associated with a good prognosis in BUC patients.

**Fig. 11:**
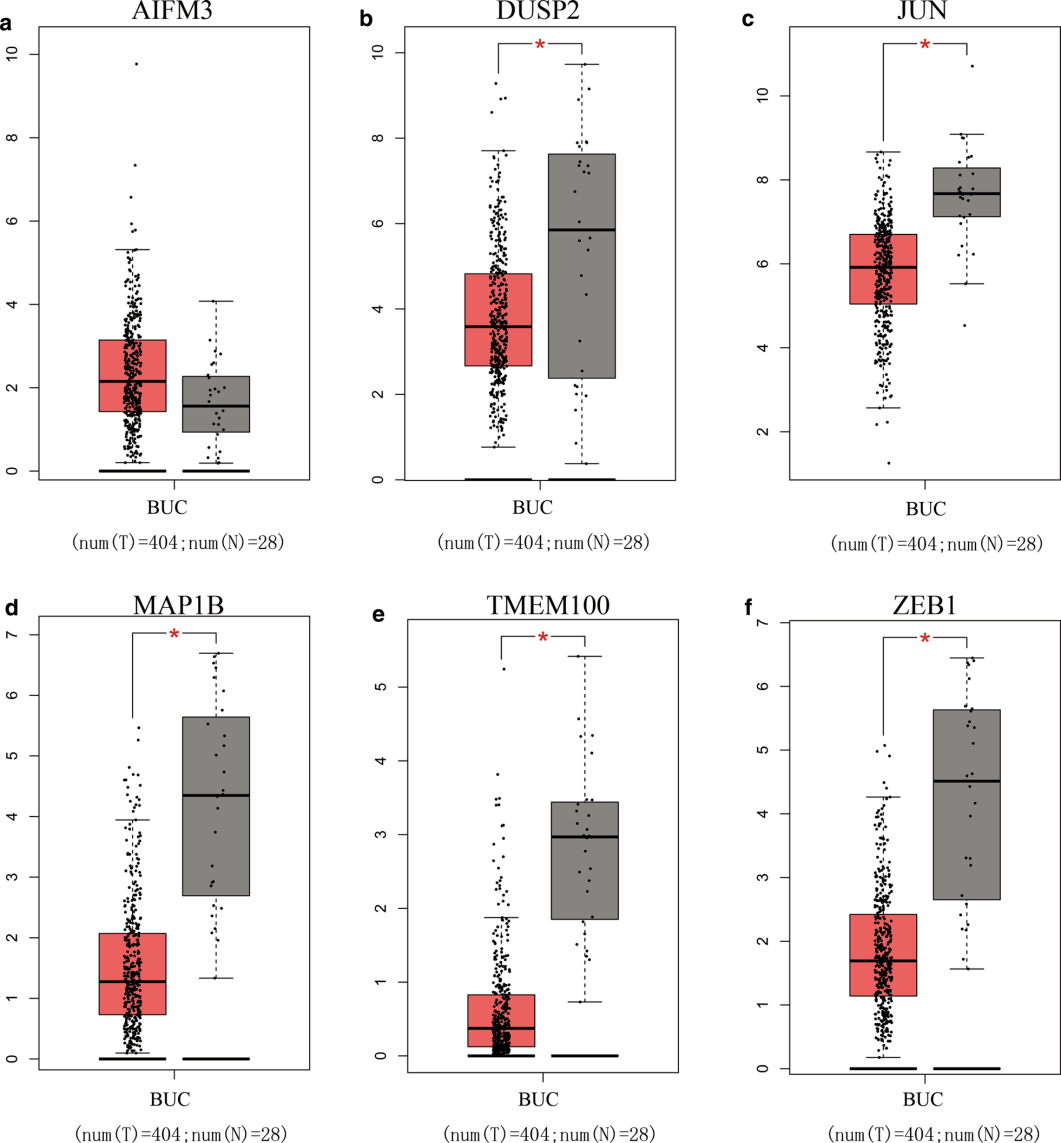
Differential expression of the six independent prognostic mRNAs (**a** for AIFM3, **b** for DUSP2, **c** for JUN, **d** for MAP1B, **e** for TMEM100 and **f** for ZEB1) in human BUC and their normal bladder controls analyzed using GEPIA. The red and gray boxes represent BUC and normal tissues respectively. (BUC, bladder urothelial carcinoma. *num* number, *T* tumor, *N* normal. *P < 0.05 and was considered statistically significant

**Fig. 12:**
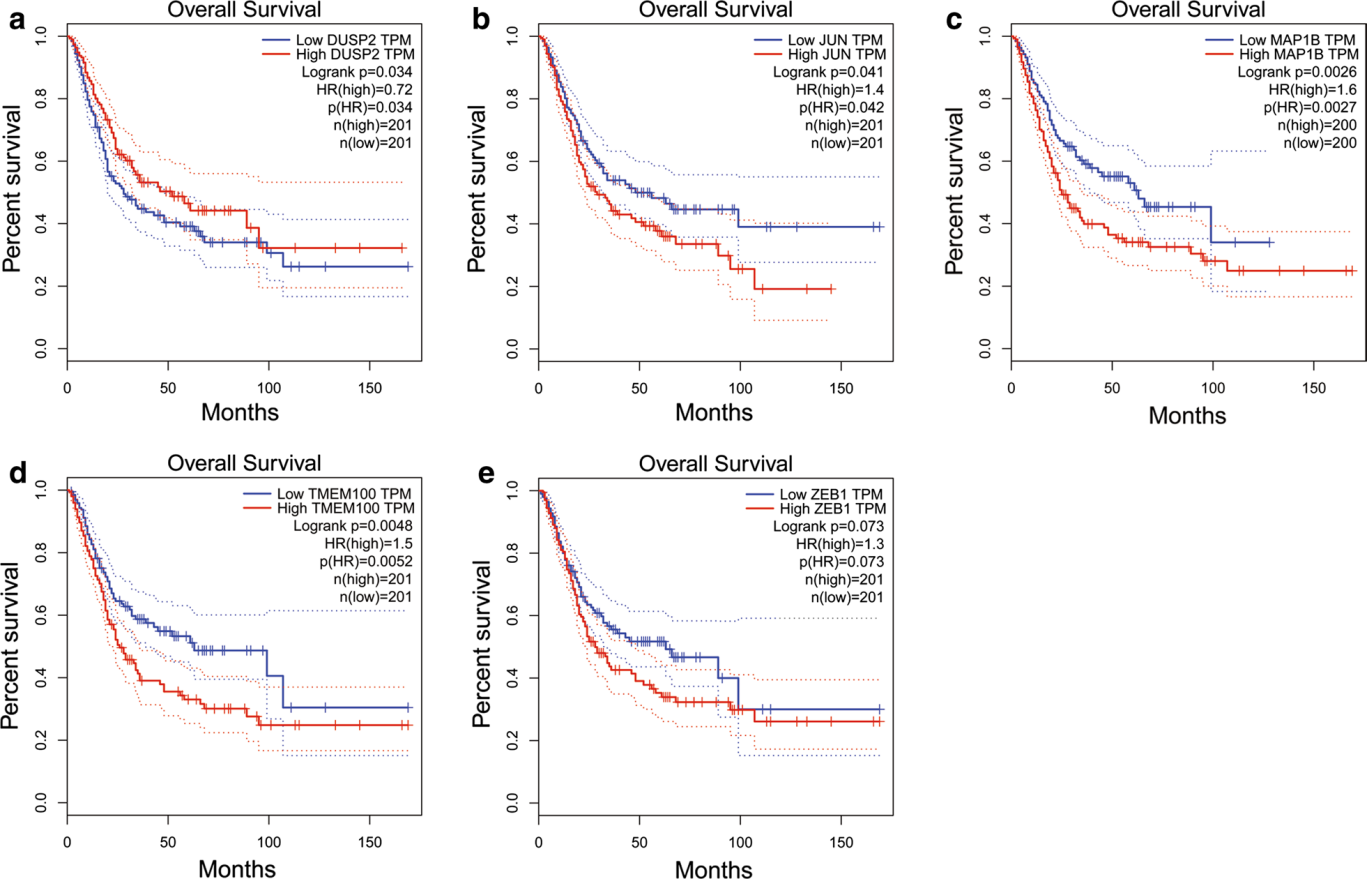
Survival analysis of the five-independent prognostic lncRNAs (**a** for DUSP2, **b** for JUN, **c** for MAP1B, **d** for TMEM100 and **e** for ZEB1) in BUC patients analyzed using GEPIA. The median expression levels of the five-independent prognostic lncRNAs were set as the thresholds for demarcating high and low-expression cohorts. Log Rank was used and P < 0.05 was considered statistically significant. *TPM*  transcripts per million. Horizontal axis: overall survival time: months, Vertical axis: survival

Similar to the lncRNAs, 6 independent prognostic mRNAs (ZEB1, TMEM100, MAP1B, DUSP2, JUN, and AIFM3) in the ceRNA network were also analyzed using GEPIA. The six mRNA expression levels are shown in Fig. 11a–f. ZEB1, TMEM100, MAP1B, DUSP2 and JUN were down-regulated with statistical significance, while AIFM3 was up-regulated without statistical significance in BUC. The correlation with overall survival of these 5 mRNAs, ZEB1, TMEM100, MAP1B, DUSP2, JUN, and AIFM3 was also estimated using GEPIA. The results are shown in Fig. 12a–e. Four mRNAs (TMEM100, MAP1B, DUSP2, and JUN) except ZEB1 (P = 0.073) had statistical significance with OS in BUC patients. Lower expression of JUN, MAP1B, and TMEM100 was associated with a good prognosis, while lower expression of DUSP2 was associated with a poor prognosis.

### Correlation of the independent prognostic factors; lncRNAs and mRNAs

**Fig. 13:**
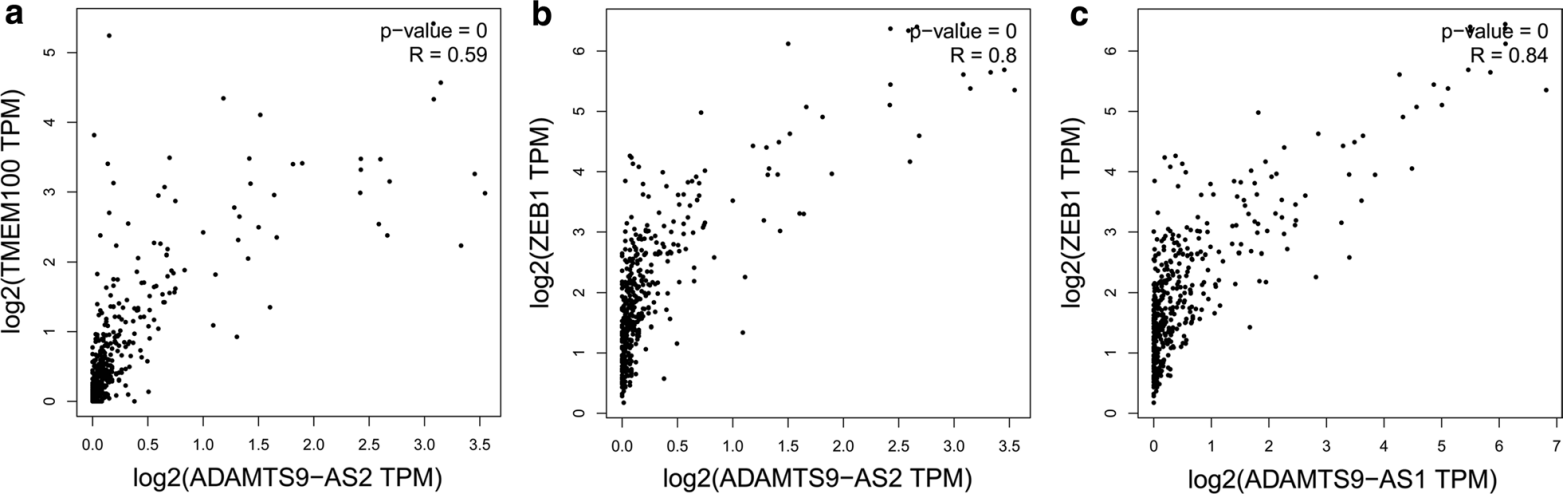
Correlation of the independent prognostic lncRNAs and mRNAs (**a** for ADAMTS9-AS2 and TMEM100, **b** for ADAMTS9-AS2 and ZEB1, **c** for ADAMTS9-AS1 and ZEB1). R values greater than 0.4 and P-values < 0.05 were considered statistically significant

With regards to the lncRNA and mRNA independent prognostic factors involved in ceRNA network, we observed that ADAMTS9-AS1 interacted with ZEB1 through hsa-mir-96, and ADAMTS9-AS2 interacted with three DEmRNAs (TMEM100, DUSP2, and ZEB1) through seven different DEmiRNAs (hsa-mir-96, hsa-mir-372, hsa-mir-183, hsa-mir-200a, hsa-mir-141, hsa-mir-373, and hsa-mir-205). Pearson correlation analysis was performed to verify the correlation of the independent prognostic lncRNA and mRNA factors. The results are shown in Fig. 13 (a for ADAMTS9-AS2 and TMEM100, B for ADAMTS9-AS2 and ZEB1, C for ADAMTS9-AS1 and ZEB1). We found that ADAMTS9-AS1 had a strong positive correlation with ZEB1, and ADAMTS9-AS2 had a strong positive correlation with ZEB1 and TMEM100. ADAMTS9-AS1 and ADAMTS9-AS2 interacted with ZEB1 and TMEM100 during BUC development. At present, no studies have demonstrated the relationship between ADAMTS9-AS1 and ZEB1, or ADAMTS9-AS2 and ZEB1 or TMEM100 in cancer.

## Discussion

In this study, five DElncRNA factors (HCG22, ADAMTS9-AS1, ADAMTS9-AS2, AC078778.1, and AC112721.1) in the ceRNAs network were identified as independent prognostic factors for OS in BUC patients. HCG22 expression levels have been reported to be down-regulated in oral cancer and its low expression was associated with poor survival in a recent study based on TCGA data analysis [22]. Lu et al. [23] investigated HCG22 expression levels in 20 oral cavity and oropharyngeal squamous cell carcinoma (OSCC) samples and 10 control samples by qRT-PCR. They demonstrated that HCG22 was downregulated in OSCC tissues compared to controls, while no association was observed between HCG22 expression levels and overall survival. ADAMTS9-AS1 (ADAMTS9 antisense transcript), is a novel lncRNA without any functional annotation but could interact with two RNA-binding proteins, DCGR8 and FUS [24]. lncRNAs play an important role in cancer mainly via their associations with RNA-binding g proteins, which include HOTTIP, MaLAT1, H19, and HOTAIR. They participate in several biological pathways involved in cell differentiation and proliferation, apoptosis and tumorigenesis by interacting with RNA-binding proteins in hepatocellular carcinoma [25]. We hypothesized that ADAMTS9-AS1 may play a role in the development of cancer. Wang et al. [26] found that ADAMTS9-1 and ADAMTS9-2 expression levels were decreased in malignant epithelial ovarian cancer tissues compared to normal ovary tissues and benign ovarian cysts using lncRNA and mRNA microarray analysis. These results were confirmed using 8 normal ovarian, 17 benign ovarian cysts and 15 malignant epithelial ovarian cancer samples by qPCR assays. Low ADAMTS9-AS2 levels were found to be a significant independent predictor of poor survival in glioma patients [27]. Liu et al. [28] suggested that lncRNA ADAMTS9-AS2 could suppress cancer progression by inhibiting miR-223-3p and activating TGFBR3. Additionally, increased ADAMTS9-AS2 levels could reduce lung cancer tumor size and improve OS. However, to date, no studies have been performed to determine the role of AC078778.1 and AC112721.1 in cancer.

miRNAs are involved in multiple roles during carcinogenesis. In this study, we found two independent prognostic DEmiRNA factors (hsa-mir-141 and hsa-mir-145) that were involved in the ceRNA network. miR-141 was found to be up-regulated in malignant bladder tissue samples compared to healthy tissues and was a favorable prognostic biomarker [29]. microRNA-141 (hsa-mir-141) has been shown to exert a regulatory role during epithelial to mesenchymal transition process, and its expression levels have been associated with tumorigenicity and invasiveness in several human cancers. hsa-mir-141 has been shown to be associated with the development of certain epithelial cancer cell types, including prostate [30], colorectal [31] and breast cancer [32]. Huang et al. [33] demonstrated that miR-141 could inhibit gastric cancer cell proliferation and tumor growth, while low miR-141 levels were associated with poor prognosis. Another study on gastric cancer suggested that miR-141 could play an important anti-tumor role by interacting with MEG3 and targeting E2F3 during gastric cancer pathogenesis and may be a therapeutic target. miR-145 has been frequently observed to be down-regulated in cancers and restoration of miR-145 levels suppressed cancer cell invasion by reversing the EMT phenotype [34]. Tan et al. [35] demonstrated that TUG1 promoted bladder cancer cell metastasis and radio-resistance by negatively regulating miR-145 expression.

In the present study, 6 prognostic DEmRNA factors (ZEB1, TMEM100, MAP1B, DUSP2, JUN, and AIFM3) were involved in the ceRNAs network and functioned as independent prognostic factors for OS in BUC patients. Several studies have demonstrated that ZEB1 was significantly overexpressed in bladder cancer tissues compared to normal healthy adjacent tissues [36]. Li et al. [37] reported that ZEB1 was significantly overexpressed in bladder cancers compared to normal tissues, and played a crucial role during VM formation, and was closely associated with invasion, metastasis and poor prognosis of malignant tumors [38, 39]. Transmembrane protein 100 (TMEM100), located at 17q32, was first identified as a transcript in the mouse genome. Han et al. [32] found that TMEM100 could function as a tumor suppressor by inhibiting the growth and metastasis of non-small-cell lung cancer via the inhibition of the TNF pathway. Low TMEM100 expression levels were associated with poor prognosis. Similar results have been reported for hepatocellular carcinoma [40]. MAP1B, which encodes for the microtubule-associated protein 1B (MAP1B), is one of the main cytoskeletal proteins. Several studies have demonstrated that MAP1B plays an important role in a number of cellular processes, including synaptic transmission, autophagy, and cancer [41–43]. DUSP2 is a member of the class 1 DUSP family of proteins and is localized in the nucleus. DUSP2 levels are significantly decreased in bladder cancer and low expression of DUSP2 is correlated with poor prognosis [44]. JUN (C-Jun, AP-1 transcription factor subunit) is specifically phosphorylated by JNK and plays a central role in the AP-1 complex. It is involved in cellular DNA damage response by regulating the expression of several genes [45, 46]. c-Jun is a proto-oncogene and is involved in transformation and tumor development [47, 48]. AIFM3 (apoptosis-inducing factor mitochondria associated 3) is a gene with homology to apoptosis-inducing factor (AIF). AIF induces apoptosis in a caspase-dependent manner. AIFM3 was observed to be highly expressed in breast cancer tissues and associated with shorter overall survival and disease-free survival [49].

BUC specific DEmRNA pathways were assessed using KEGG pathway analysis. Our results demonstrated that the majority of DEmRNAs were frequently enriched for cancer-related pathways. Of these, the neuroactive ligand-receptor interaction signaling pathway has been reported to be associated with the progression of renal cell carcinoma in bioinformatics studies [50]. In addition, previous studies have suggested that cAMP-related signaling could control apoptosis induction and cell growth [51, 52], while another study demonstrated that cAMP was an inhibitor of cell cycle progression and apoptosis in gastric cancer cells [53]. Zhang et al. [54] showed that the ECM–receptor interaction pathway played a significant role in tumor progression and metastasis. We found several overlapping DEmRNAs that were involved in multiple pathways, such as SLC8A1, CAMK2B, and CACNA2D1, and have been demonstrated to play important roles in cancer pathogenesis. Muñoz reported that lower levels of SLC8A1, which was at least partly mediated by miR-223, was associated with reduced calcium and apoptosis levels in penile carcinoma [55]. Based on an integrative meta-analysis, CAMK2B was found to be associated with the development of cancer cachexia [56]. Recently, Feng et al. [57] assayed 15 colorectal cancer tissues and 10 paracancerous tissues using microarrays and found that CAMK2B was involved in the progression of Fusobacterium nucleatum-induced colorectal cancer. Another study also reported that CAMK2B played an important role in glioblastoma multiforme using bioinformatics analysis on publicly available datasets [58]. High expression levels of CACNA2D1 in epithelial ovarian cancers were significantly correlated with histological subtypes, advanced FIGO stages and tumor differentiation [59]. In addition, the majority of PLB4 hotspot mutations are gain-of function mutations that have been demonstrated to be involved in uveal melanoma tumorigenesis by activating the same signaling pathway [60].

In terms of GO functional enrichment analysis, we found that DEmRNAs were significantly enriched for the regulation of multiple processes, such as binding, protein binding, receptor binding, ion channel activity, and endopeptidase activity. Interestingly, several studies have reported that these functions overlapped in different cancers. Several studies have demonstrated that knocking down ezrin and P65 expression induces tumor metastasis in different cancers [61, 62]. Furthermore, Tang et al. [63] demonstrated that ezrin and P65 were physically associated with one another. We hypothesize that the interaction between ezrin and P65 is associated with the activation of the NF-κB pathway leading to breast cancer metastasis. A previous study suggested that Estrogen receptor β could increase the levels of miR-92a by binding to the estrogen-response-element (ERE) leading to a decrease in DAB2IP tumor suppressor expression to ultimately promote bladder cancer growth and invasion [64]. Numerous studies have demonstrated that ion channels play an important role in tumorigenesis and progression, such as inducing neo-angiogenesis [65], apoptosis resistance [66], proliferative potential [67] as well as cell migration and invasiveness [68, 69]. In terms of endopeptidase activity, Zhu et al. [70]. demonstrated that asparaginyl endopeptidase (AEP) was highly expressed in tissues and ascites of patients with epithelial ovarian cancer and promoted tumor growth and progression both in vivo and in vitro.

We then constructed a PPI network to identify hub DEmRNAs. Proteins that corresponded to genes were used to build the PPI network, and the top 30 DEmRNAs with a high degree were selected. KEGG pathway analysis of these 30 DEmRNAs was performed using the “clusterProfiler” package. The results showed that 21 of these 30 DEmRNAs were enriched for “viral carcinogenesis” and “transcriptional dysregulation in cancer”. The 21 identified DEmRNAs may play an important role in cancer. For example, IL6, HIST1H3C, and HIST1H3G, which were classified with high degrees are present in pathways associated with transcriptional dysregulation in cancer. In addition, these three genes have been previously reported to be closely associated with tumorigenesis and development [71–73].

In terms of correlation analysis, a positive correlation between ZEB1 and ADAMTS9-AS1-AS2 and TMEM100 was observed using the Pearson correlation statistic based on the expression levels of these genes. The correlation between these four genes may play an important role in the initiation and progression of BUC. Only a few studies on their interactions have been published in public databases, such as Pubmed and Embase.

Several limitations of the present study should be stated. First, the number of normal bladder tissues (19 samples) was limited and may have compromised the reliability of our results. Second, BUC patient information from TCGA was not validated using experimental procedures. Third, we only investigated the ceRNAs network associated with lncRNAs, miRNAs, and mRNAs, and did not include other regulatory models. Finally, several novel lncRNAs with significant clinical value needs to be investigated to determine their functional role and underlying mechanism during BUC carcinogenesis. We verified the independent prognostic lncRNAs and mRNAs using GEPIA and this supported our findings. Our results provide a better understanding of lncRNA-related ceRNAs and its important role in BUC. Additional experimental and clinical studies are needed to validate our findings.

## Conclusions

Five independent prognostic DElncRNAs (HCG22, ADAMTS9-AS1, ADAMTS9-AS2, AC078778.1, and AC112721.1) in the ceRNA network, two DEmiRNAs (hsa-mir-141 and hsa-mir-145) and six DEmRNAs (ZEB1, TMEM100, MAP1B, DUSP2, JUN, and AIFM3) were identified to be closely associated with BUC pathogenesis. Two independent prognostic lncRNAs (ADAMTS9-AS1 and ADAMTS9-AS2) and four independent prognostic mRNAs (DUSP2, JUN, MAP1B, and TMEM100) were validated using GEPIA. ADAMTS9-AS1 and ADAMTS9-AS2 interacting with ZEB1 and TMEM100 may play significant roles in BUC development. Key hub DEmRNAs and their relevant pathways may play central or significant roles in BUC tumorigenesis and progression.

## Supplementary information

**Additional file 1: Figure S1.** A. Heatmap of DElncRNAs. B. Heatmap of DEmRNAs. C. Heatmap of DEmiRNAs.

**Additional file 2: Figure S2.** The network of significant-top 15 KEGG pathways enriched in the DEmRNAs. Red nodes represent increased expression levels, while blue nodes represent decreased expression levels. Triangle nodes represent DEmRNAs; Green ellipse nodes represent enrichment pathways. Gray edges indicate mRNAs involved in the pathway.

**Additional file 3: Figure S3.** The top 30 hub DEmRNAs identified using the ranking method of degree.

**Additional file 4: Figure S4.** (A) KEEG pathways enriched using the hub DEmRNAs; (B) Network between hub DEmRNAs and KEGG pathways enriched by hub DEmRNAs. The red nodes represent increased expression levels, while the blue nodes represent decreased expression levels. Triangle nodes represent DEmRNAs; Green ellipse nodes represent enrichment pathways. Gray edges indicate mRNAs involved in the pathway.

**Additional file 5: Figure S5.** Heatmap of independent prognostic factors involved in the ceRNA network (A for DElncRNA, B for DEmiRNA and C for DEmRNA).

**Additional file 6.** R software, version 3.4.3.

## Data Availability

The authors declare that the data supporting the findings of this study are available within the article. The R script, which was used to generate figures and reproduce key findings in this study, was stored as Additional file 6.

## Abbreviations

BC: bladder cancer
BUC: bladder urothelial carcinoma
lncRNA: long non-coding RNA
TCGC: The Cancer Genome Atlas
FC: fold change
OS: overall survival
PPI: protein–protein interaction
miRNA: microRNA
mRNA: message RNA
MRE: microRNA response element
ceRNA: competing endogenous RNA
DEmRNAs: differentially expressed mRNAs
DElncRNAs: differentially expressed lncRNAs
DEmiRNAs: differentially miRNAs
FDR: false discovery rate
GEPIA: Gene Expression Profiling Interactive Analysis
GTEx: Genotype-Tissue Expression
