# Supplementary figures and images for: Identification and analysis of long non-coding RNA related miRNA sponge regulatory network in bladder urothelial carcinoma
Source: Cancer Cell Int. 2019 Dec 3;19:327. doi: 10.1186/s12935-019-1052-2 (PMC6892182; doi:10.1186/s12935-019-1052-2)

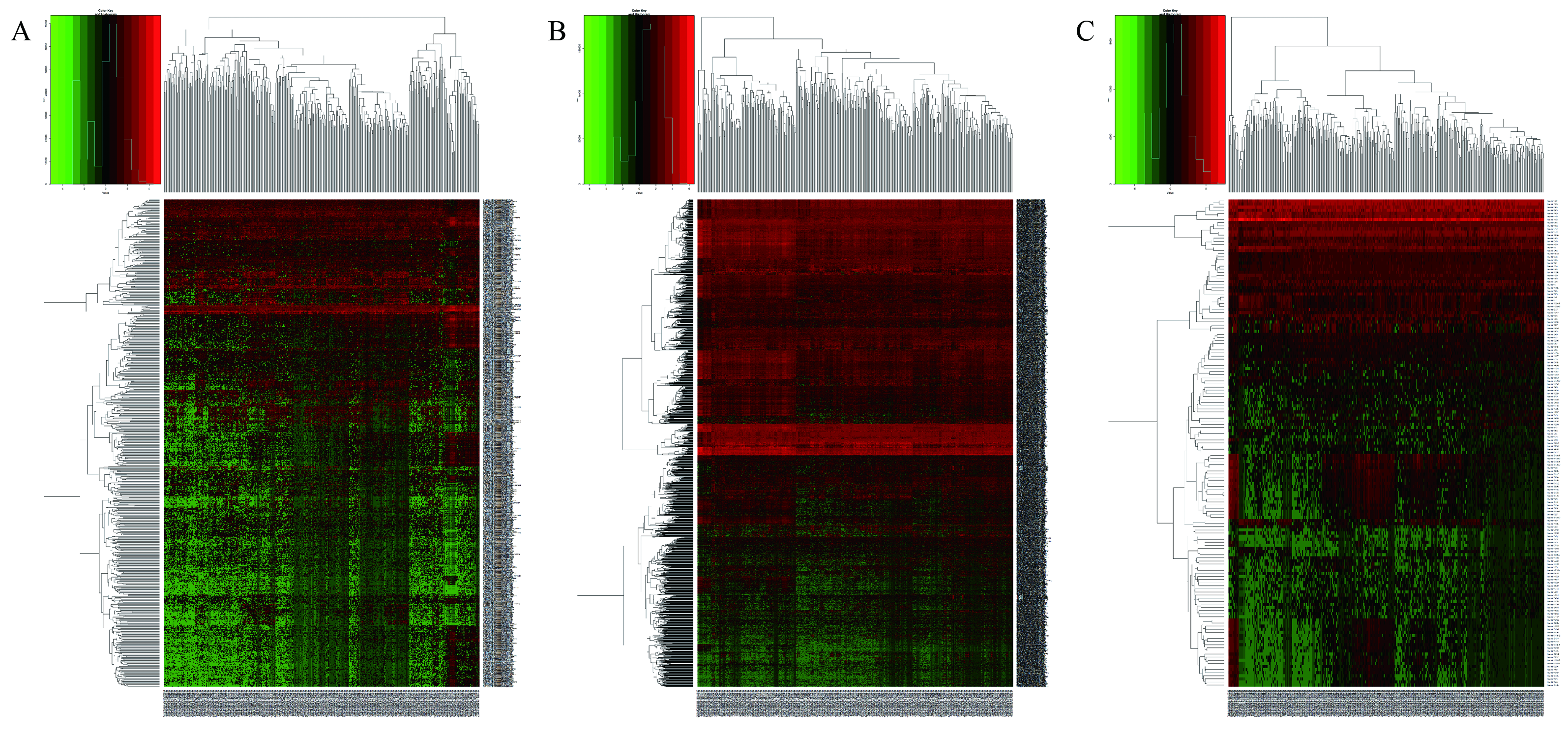

Supplement: Supplementary file 1 — Additional file 1: Figure S1. A. Heatmap of DElncRNAs. B. Heatmap of DEmRNAs. C. Heatmap of DEmiRNAs. [file 12935_2019_1052_MOESM1_ESM.tif]

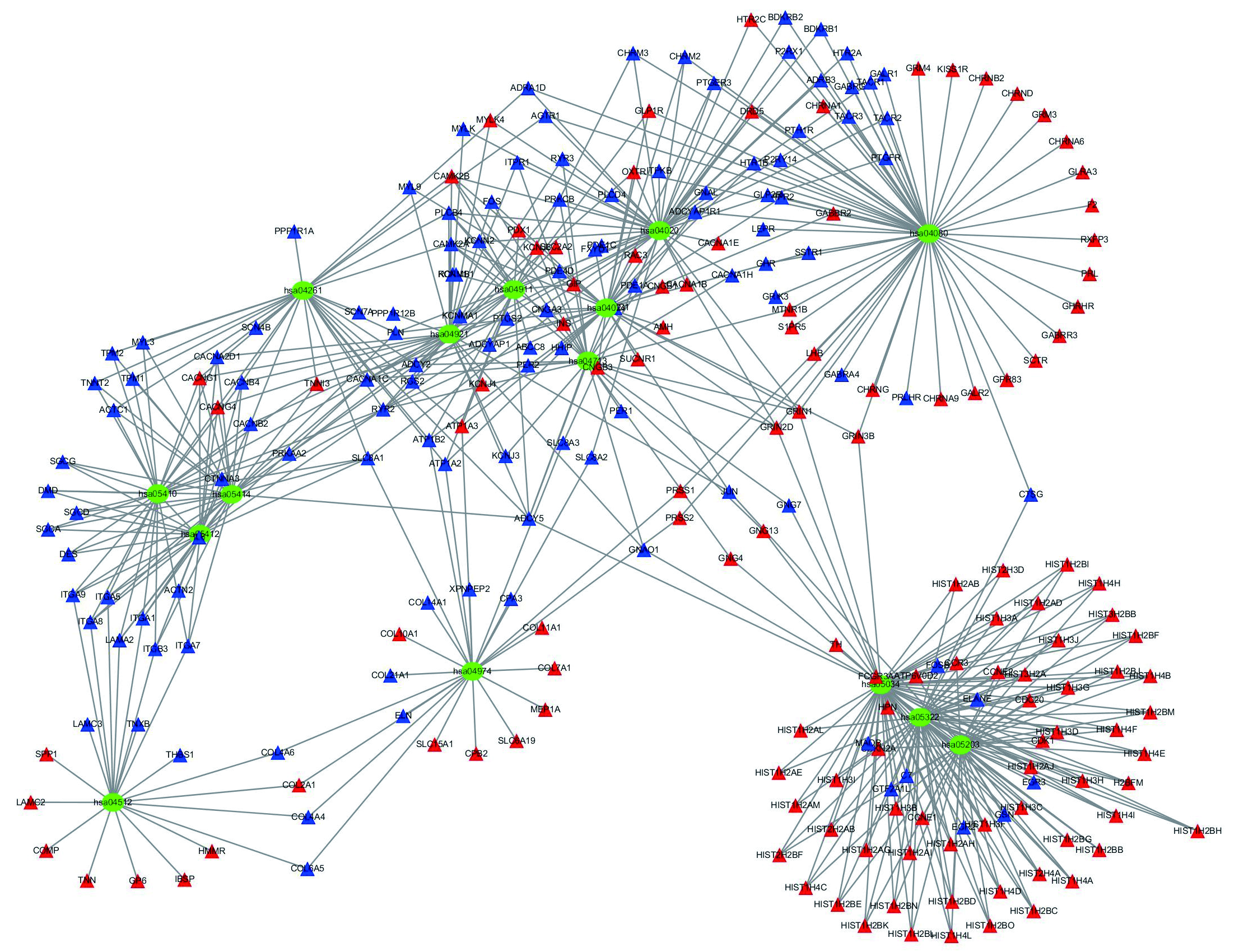

Supplement: Supplementary file 2 — Additional file 2: Figure S2. The network of significant-top 15 KEGG pathways enriched in the DEmRNAs. Red nodes represent increased expression levels, while blue nodes represent decreased expression levels. Triangle nodes represent DEmRNAs; Green ellipse nodes represent enrichment pathways. Gray edges indicate mRNAs involved in the pathway. [file 12935_2019_1052_MOESM2_ESM.tif]

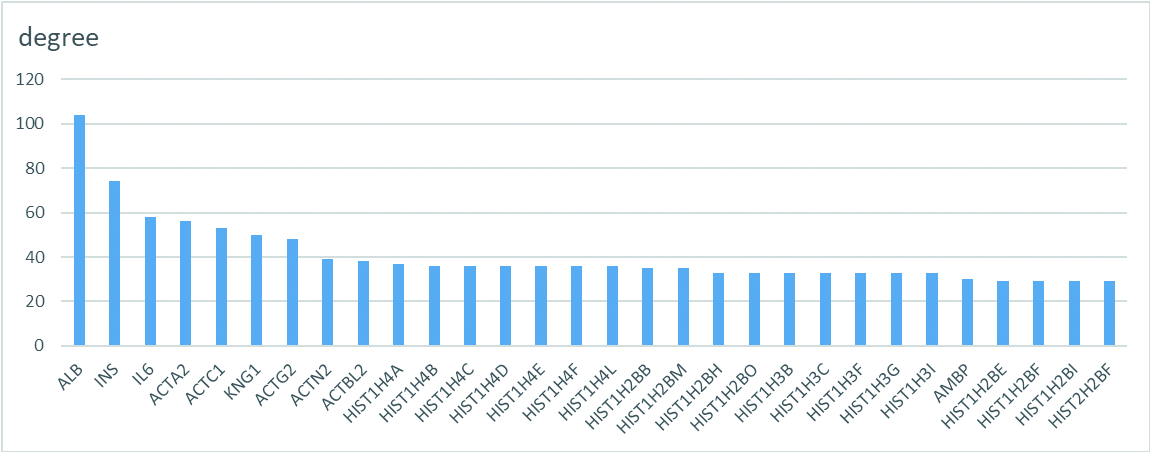

Supplement: Supplementary file 3 — Additional file 3: Figure S3. The top 30 hub DEmRNAs identified using the ranking method of degree. [file 12935_2019_1052_MOESM3_ESM.tif]

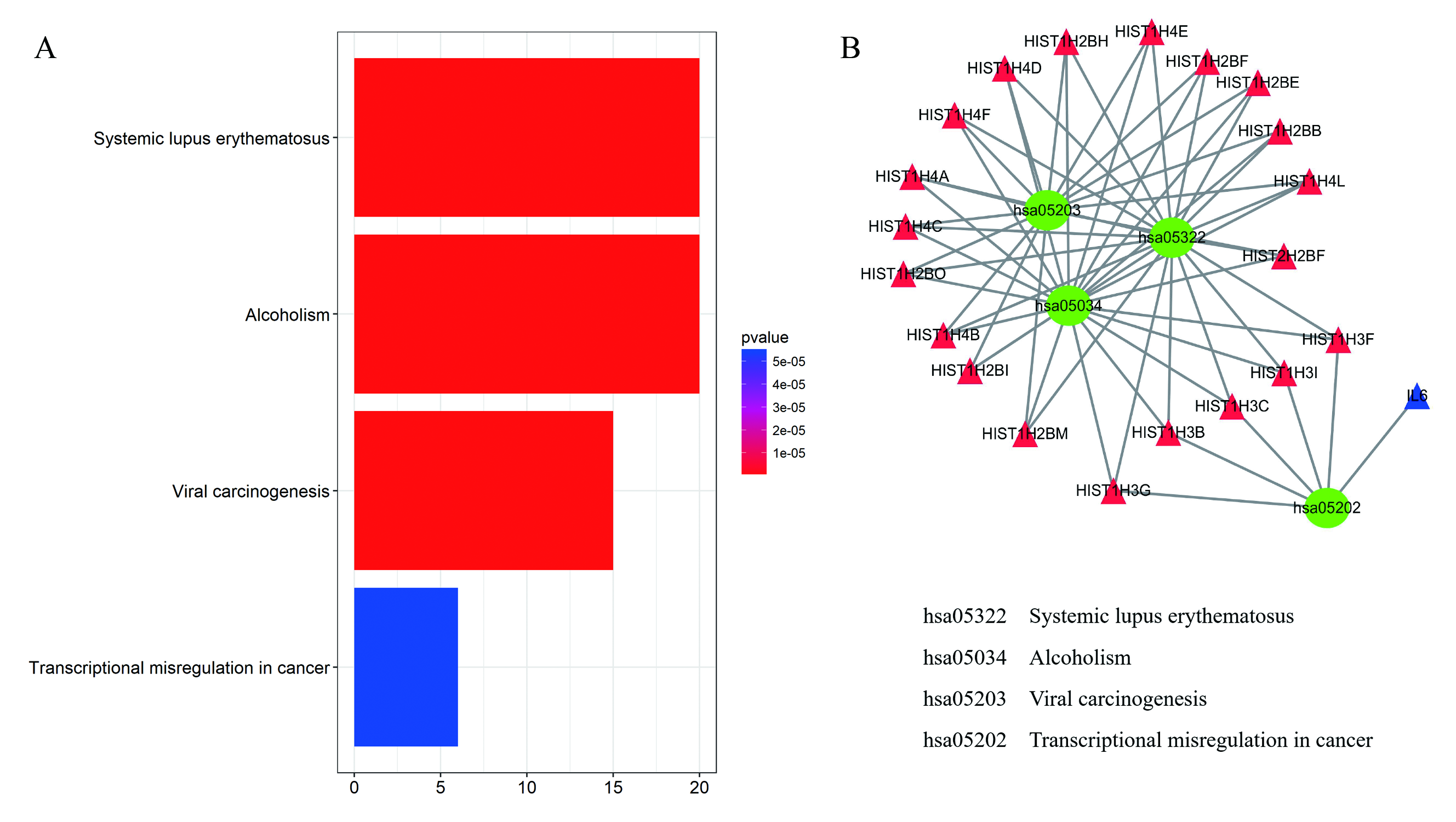

Supplement: Supplementary file 4 — Additional file 4: Figure S4. (A) KEEG pathways enriched using the hub DEmRNAs; (B) Network between hub DEmRNAs and KEGG pathways enriched by hub DEmRNAs. The red nodes represent increased expression levels, while the blue nodes represent decreased expression levels. Triangle nodes represent DEmRNAs; Green ellipse nodes represent enrichment pathways. Gray edges indicate mRNAs involved in the pathway. [file 12935_2019_1052_MOESM4_ESM.tif]

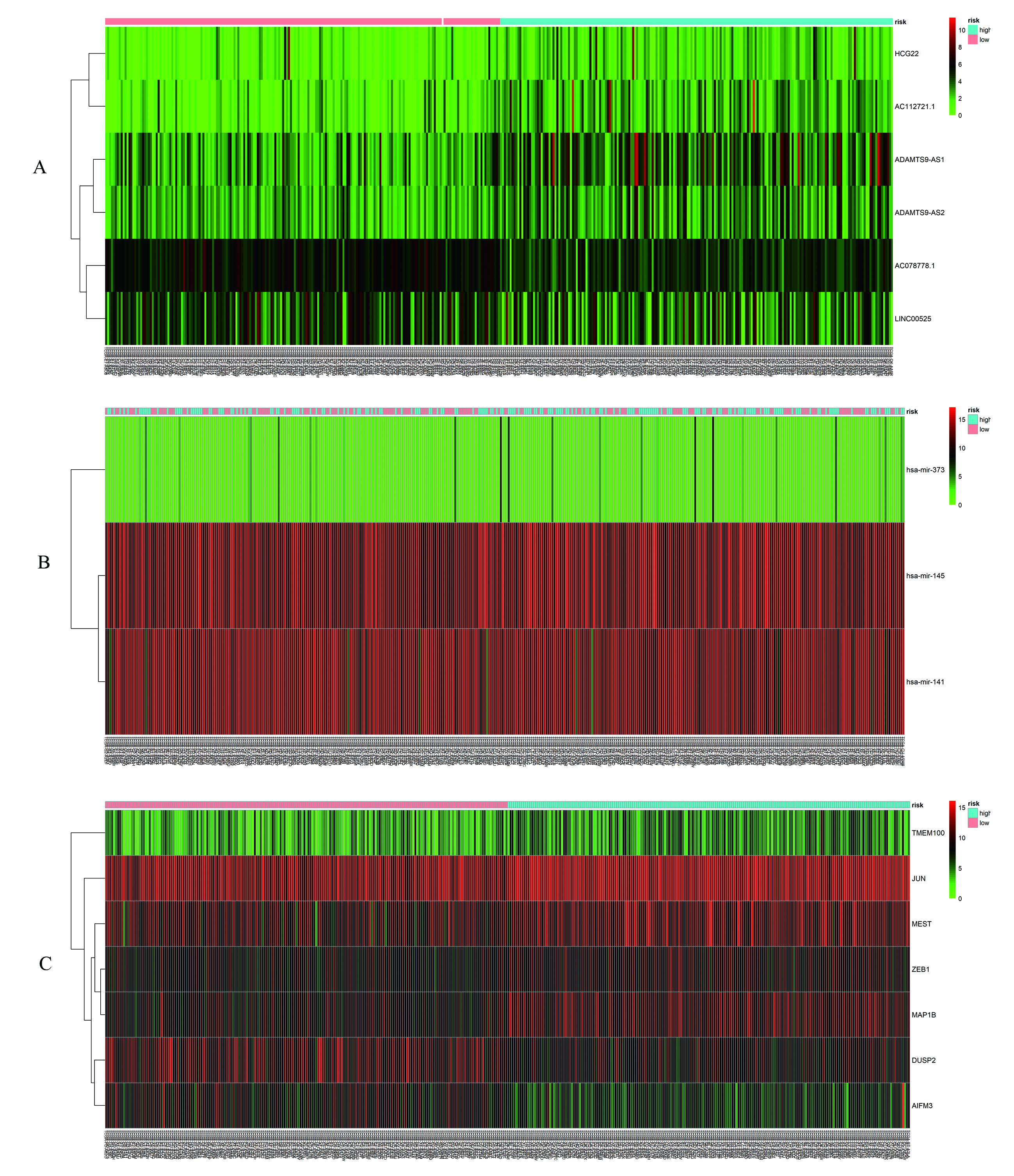

Supplement: Supplementary file 5 — Additional file 5: Figure S5. Heatmap of independent prognostic factors involved in the ceRNA network (A for DElncRNA, B for DEmiRNA and C for DEmRNA). [file 12935_2019_1052_MOESM5_ESM.tif]
